# Mitochondrion-processed *TERC* regulates senescence without affecting telomerase activities

**DOI:** 10.1007/s13238-019-0612-5

**Published:** 2019-02-20

**Authors:** Qian Zheng, Peipei Liu, Ge Gao, Jiapei Yuan, Pengfeng Wang, Jinliang Huang, Leiming Xie, Xinping Lu, Fan Di, Tanjun Tong, Jun Chen, Zhi Lu, Jisong Guan, Geng Wang

**Affiliations:** 1 MOE Key laboratory of Bioinformatics, Cell Biology and Development Center, School of Life Sciences, Tsinghua University, Beijing, 100084 China; 2 Peking University Research Center on Aging, Beijing, 100191 China; 3 Department of Biochemistry and Molecular Biology, Peking University Health Science Center, Beijing, 100191 China

**Keywords:** mitochondria, retrograde signal, nucleus, transcription regulation, non-coding RNA, telomerase

## Abstract

Mitochondrial dysfunctions play major roles in ageing. How mitochondrial stresses invoke downstream responses and how specificity of the signaling is achieved, however, remains unclear. We have previously discovered that the RNA component of Telomerase *TERC* is imported into mitochondria, processed to a shorter form *TERC-53*, and then exported back to the cytosol. Cytosolic *TERC-53* levels respond to mitochondrial functions, but have no direct effect on these functions, suggesting that cytosolic *TERC-53* functions downstream of mitochondria as a signal of mitochondrial functions. Here, we show that cytosolic *TERC-53* plays a regulatory role on cellular senescence and is involved in cognition decline in 10 months old mice, independent of its telomerase function. Manipulation of cytosolic *TERC-53* levels affects cellular senescence and cognition decline in 10 months old mouse hippocampi without affecting telomerase activity, and most importantly, affects cellular senescence in *terc*^−/−^ cells. These findings uncover a senescence-related regulatory pathway with a non-coding RNA as the signal in mammals.

**Electronic supplementary material:**

The online version of this article (10.1007/s13238-019-0612-5) contains supplementary material, which is available to authorized users.

## Introduction

Human mitochondrial dysfunctions are linked to ageing related health problems such as metabolic defects, neurodegenerative diseases, and self-immune diseases (Bishop et al., 2010; Lopez-Otin et al., 2016; Sun et al., 2016). Mutations that affect mitochondrial bioenergetics and biosynthesis have been shown to modulate signaling pathways and gene expression through mitochondrial retrograde signaling (Wallace, 2012; Guha and Avadhani, 2013). The most well studied mitochondrial retrograde signals are calcium ion and ROS (Kotiadis et al., 2014; Sullivan and Chandel, 2014; Reczek and Chandel, 2015; Gottlieb and Bernstein, 2016). A decrease in calcium import into mitochondria increases cytosolic calcium concentration, which leads to transcriptional upregulation of over 100 genes including those involved in glucose metabolism, apoptosis and tumorigenesis (Kotiadis et al., 2014; Gottlieb and Bernstein, 2016). Mitochondrion-produced ROS initiate signaling in cellular processes such as proliferation, antioxidant gene regulation, apoptosis and aging through redox mechanism (Sullivan and Chandel, 2014; Reczek and Chandel, 2015). Both Ca^2+^ and ROS, however, lack specificity as signaling molecules. How mitochondria regulate a cellular process through retrograde signaling specifically remains relatively unexplored.

The roles mitochondria play during ageing are more complicated than originally expected. Mitochondrial dysfunctions contribute to different aspects of ageing including cellular senescence, decline of stem cell activity and inflammation (Sun et al., 2016). Mitochondrial impairments trigger mitochondrial unfolded protein responses and mitophagy that also have protective effects and in turn may extend longevity in model organisms such as *C*. *elegans* (Yee et al., 2014; Schulz and Haynes, 2015; Sun et al., 2016). These processes are regulated by a complex network of signaling pathways, and the intricate interplay and co-regulation only start to unfold.

Mitochondria import a variety of cytosolic non-coding RNAs, including tRNAs, rRNAs, microRNAs and lncRNAs (Chang and Clayton, 1989; Alfonzo and Soll, 2009; Wang et al., 2010; Mercer et al., 2011; Zhang et al., 2014; Cheng et al., 2018). The import pathway is partially characterized in mammalian cells with PNPASE, a mitochondrial IMS (intermembrane space) protein, as an important regulator (Wang et al., 2010; Vedrenne et al., 2012; von Ameln et al., 2012; Sato et al., 2017). The mitochondrial functions of most imported RNAs, however, are unclear. We have previously discovered that the RNA component of Telomerase *TERC* is imported into mitochondria, processed to a shorter form *TERC-53* by mitochondrial RNASET2, and then exported back to the cytosol (Cheng et al., 2018). Cytosolic *TERC-53* levels respond to mitochondrial functions, but have no direct effect on these functions, suggesting that it could function as a mitochondrial retrograde signal (Cheng et al., 2018). Here, we show that cytosolic *TERC-53* regulates cellular senescence and is involved in cognition decline in 10 months old mouse hippocampus without affecting telomerase activity or mitochondrial functions, possibly through regulating nuclear gene expression. These findings demonstrate that a non-coding RNA functions as a specific signaling molecule, a potential general mechanism, and provide a mechanism on how mitochondria regulates cellular senescence and possibly organismal ageing in mammals.

## Results

### *TERC-53* regulates cellular senescence

**Figure 1:**
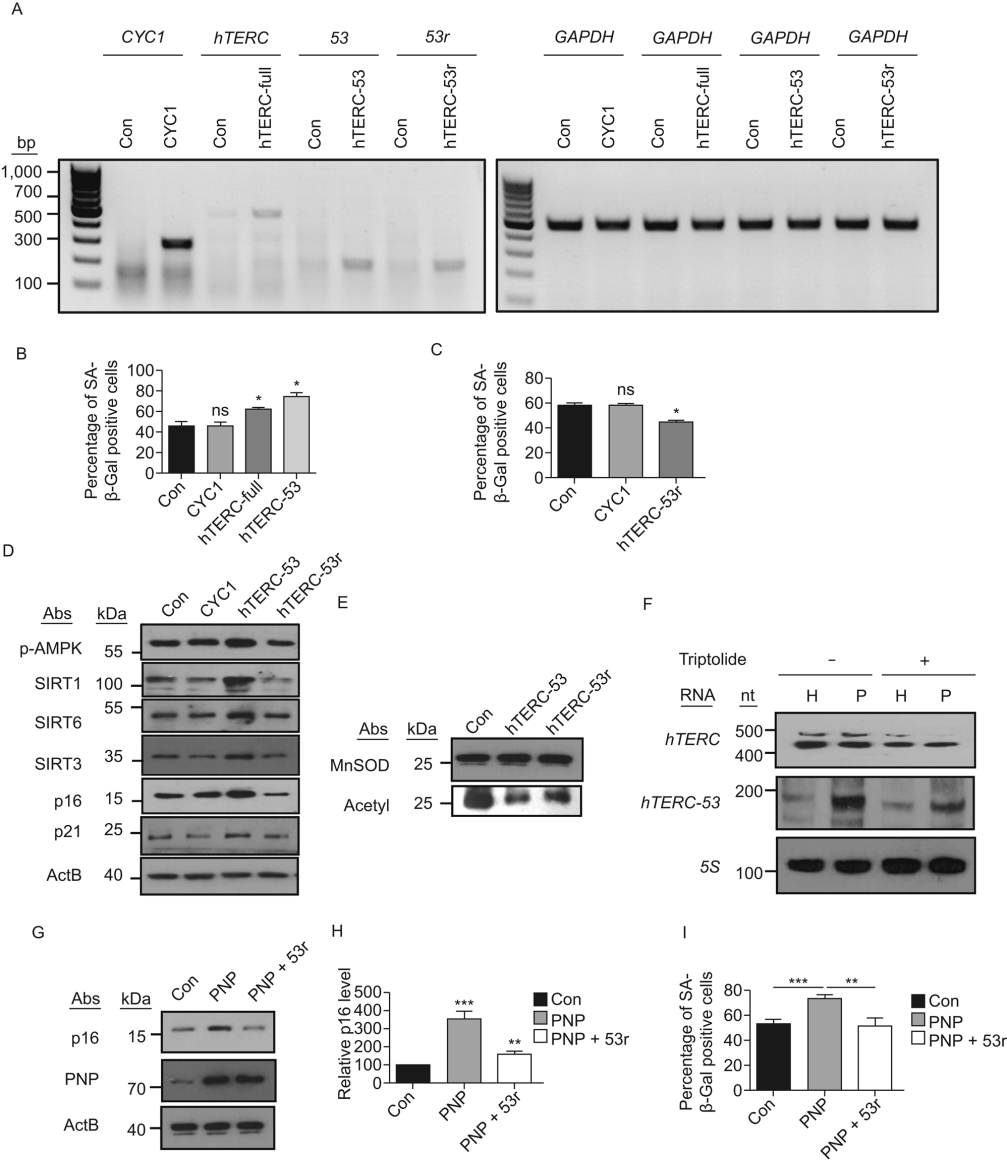
***hTERC-53*regulates cellular senescence**. (A) Total RNA isolated from 2BS cell lines generated with the empty vector (con), or the vector expressing yeast *CYC1* RNA (CYC1), full length *hTERC* (hTERC-full), *hTERC-53* (hTERC-53) or *hTERC-53r* (hTERC-53r) were used as templates for RT-PCR with primers for *CYC1*, *hTERC*, *TERC-53* (*53*), *TERC-53r* (*53r*) or *GAPDH*. (B) 2BS cells made with the empty vector (con), or the vector expressing yeast *CYC1* RNA (CYC1), full length *hTERC* (hTERC-full) or *hTERC-53* (hTERC-53) were grown to 37 PDs, and then stained for SA-β-gal. The bar graph shows the percentage of SA-β-gal positive cells. (C) 2BS cells made with the empty vector (con), or the vector expressing yeast *CYC1* RNA (CYC1) or anti-sense *hTERC-53* (hTERC-53r) were grown to 43 PDs and stained for SA-β-gal. (D) Immunoblots of the cell lysates with *hTERC-53*, *hTERC-53r* or *CYC1* overexpression. (E) Immunoblots of MnSOD (MnSOD: Manganese Superoxide Dismutase) immunoprecipitation samples from cell lysates with *hTERC-53* or *hTERC-53r* overexpression (Acetyl: acetylated MnSOD). (F) Northern blots of cytosolic *hTERC*, *hTERC-53* and *5S* rRNA in HEK cells (H), and HEK cells overexpressing PNPASE (P) with or without triptolide treatment (2 μmol/L for 3 h). (G) Immunoblots of HEK293 cells overexpressing PNPASE (PNP) or PNPASE with *hTERC-53r* (PNP + 53r) (con: HEK cells harboring the empty vector). (H) Quantification of the relative p16 level in panel (G) (*n* = 3). (I) Percentage of SA-β-gal positive cells after H_2_O_2_ treatment and 3 days’ recovery. Statistical comparisons are performed using unpaired *t*-tests; *P < 0.05, **P < 0.01, ***P < 0.001, ****P < 0.0001. Data are presented as mean ± standard error of the mean (s.e.m.)

We have previously shown that the RNA component of Telomerase *TERC* is imported into mitochondria, processed to a shorter form *TERC-53*, and then exported back to the cytosol (Cheng et al., 2018). In the cells, *TERC-53* is localized predominately in the cytosol. Cytosolic *TERC-53* level responds to mitochondrial functions, but has no direct effect on these mitochondrial functions (Cheng et al., 2018). To investigate the function of cytosolic *TERC-53*, we stably overexpressed the RNA in 2BS (a primary strain of human embryonic lung fibroblast) cells with *H1* promoter (Figs. 1A and S1A). Consistent with the previous results (Cheng et al., 2018), *hTERC-53* overexpression led to a two fold increase of the cytosolic *hTERC-53* level, but had no effect on *hTERC* level (Fig. S1A). *hTERC-53* overexpressing cells showed a significantly faster senescence rate (Figs. 1B and S1D). Full length *hTERC* overexpressing cells also showed a similar phenotype, even though to a lesser extent (Fig. 1B), possibly the result of *hTERC-53* accumulation due to overexpression of the full length RNA (Fig. S1B). An impact on cellular senescence, however, could be the results of many factors and the *hTERC-53* effect could be indirect. To explore these alternatives, we constructed a stable cell line expressing anti-sense *hTERC-53* (*hTERC-53r*). Expression of *hTERC-53r* significantly reduced the cytosolic *hTERC-53* level, but had no effect on *hTERC* level (Fig. S1C), leading to a slowdown of the senescence rate (Figs. 1C and S1E).

Expression levels and modifications of cellular senescence markers were examined in the cells. An increase of p16 protein level was observed in *hTERC-53* overexpressing cells (Fig. 1D). Interestingly, the expression levels of many pro-longevity factors such as SIRT proteins were also upregulated and the inhibitory modification of MnSOD was removed in *hTERC-53* overexpressing cells, suggesting a compensatory response (Fig. 1D and 1E). Expression of *hTERC-53r* led to minor downregulation of the expression levels of the senescence markers such as p-AMPK and p16, and removal of the inhibitory modification of MnSOD (Fig. 1D and 1E). It remains to be elucidated how expression of *hTERC-53* and its antisense RNA could lead to the similar effect on MnSOD modification. However, overexpression and knockdown having similar effect is not uncommon with protein expressing genes (Phadke et al., 2011); Nicholls et al., 2012); it could also be the case with non-coding RNA regulation. These results also indicate that the role of *TERC-53* during cellular senescence is more regulatory than causative.

It has been shown previously that PNPASE overexpression exacerbates cellular senescence (Sarkar et al., 2003). PNPASE is involved in *TERC* trafficking into mitochondria where it is processed to *TERC-53* by RNASET2, and PNPASE overexpression leads to an increase of cytosolic *hTERC-53* level (Liu et al., 2017; Cheng et al., 2018). The increase of cytosolic *hTERC-53* level by PNPASE overexpression is not due to a drop in the RNA degradation rate, as cytosolic *hTERC-53* showed a faster degradation rate in PNPASE overexpression cells (Fig. 1F). To examine whether cytosolic *TERC-53* is involved in PNPASE’s function in cellular senescence, *hTERC-53r* was expressed in the PNPASE overexpressing cells. The increase of senescence rate and p16 level caused by PNPASE overexpression was mostly reversed by *hTERC-53r* (Fig. 1G–I), further proving the involvement of *TERC-53* in cellular senescence.

### *TERC-53* functions independent of telomerase activity

**Figure 2:**
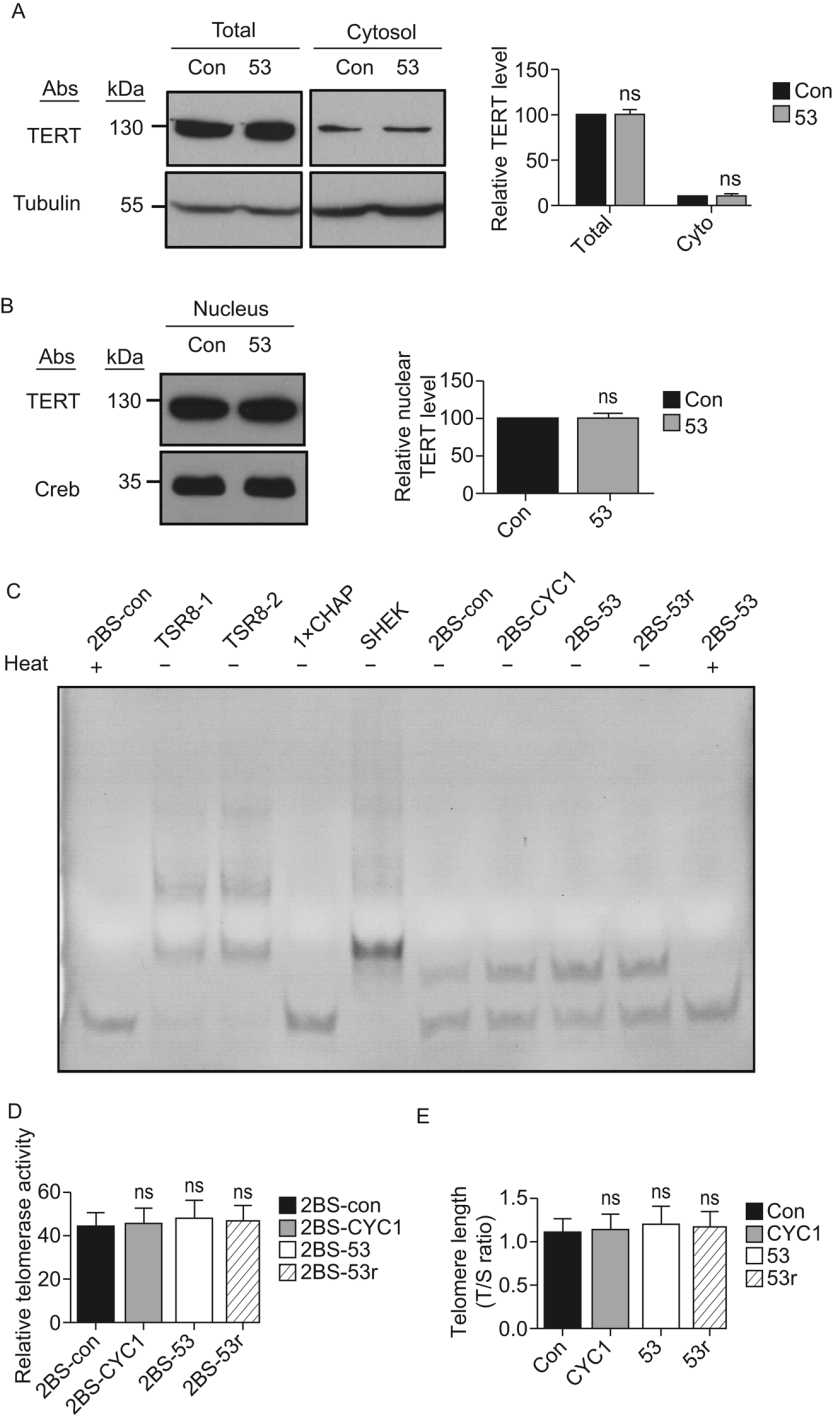
***hTERC-53*expression does not have a clear effect on the level of telomerase protein TERT or telomerase activity**. (A and B) TERT immunoblots of different fractions of HEK293 cells overexpressing *hTERC-53* (53) or with the control vector (con). β-Tubulin was used as a loading control for total lysate and the cytosol and Creb for the nucleus. The graphs on the right (panel (A)) and the bottom (panel (B)) show the quantification of relative TERT levels. (C) Telomere-repeat amplification assay of telomerase activity in 2BS cells expressing yeast *CYC1* RNA (CYC1) or anti-sense *hTERC-53* (53r), overexpressing *hTERC-53* (53), or generated with the vector only (con). HEK293 (HEK) cell lysate was used as a positive control. 1× CHAPS lysis buffer was used as primer-dimer/PCR contamination control. TSR8 template was used as telomerase quantitation control. Heat-treated extracts were used as negative controls. (D) Quantification of the telomerase activity. (E) Comparison of the telomere length in 2BS cells expressing yeast *CYC1* RNA (CYC1) or anti-sense *hTERC-53* (53r), overexpressing *hTERC-53* (53), or generated with the vector only (con). Statistical comparisons are performed using unpaired *t*-tests; *P < 0.05, **P < 0.01, ***P < 0.001, ****P < 0.0001. Data are presented as mean ± standard error of the mean (s.e.m.)

Since *TERC* is the essential RNA component of telomerase (Gall, 1990), we investigated whether *TERC-53* functions by interfering with telomerase activity. The level, localization and activity of telomerase protein TERT were examined in *hTERC-53* overexpressing and control cells. No significant difference of TERT level was observed in either the cytosol or the nucleus between *hTERC-53* overexpressing cells and the control cells (Fig. 2A and 2B). Cell lysates from 2BS cells expressing *CYC1*, *hTERC-53r* or overexpressing *hTERC-53* all showed weak but similar level of telomerase activity (Fig. 2C and 2D). Telomere length was also examined in the 2BS cell lines, and no significant difference was observed (Fig. 2E).

**Figure 3:**
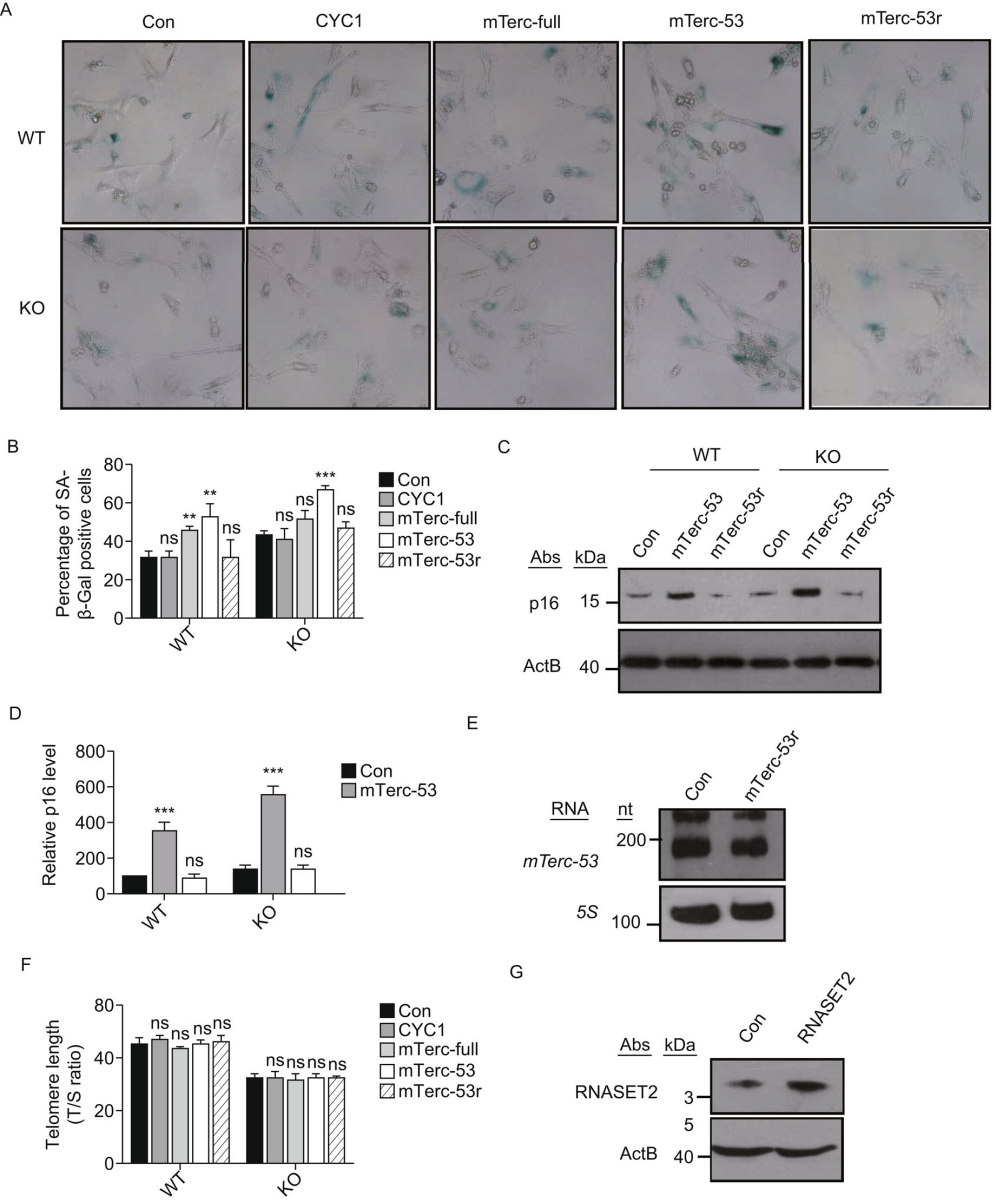

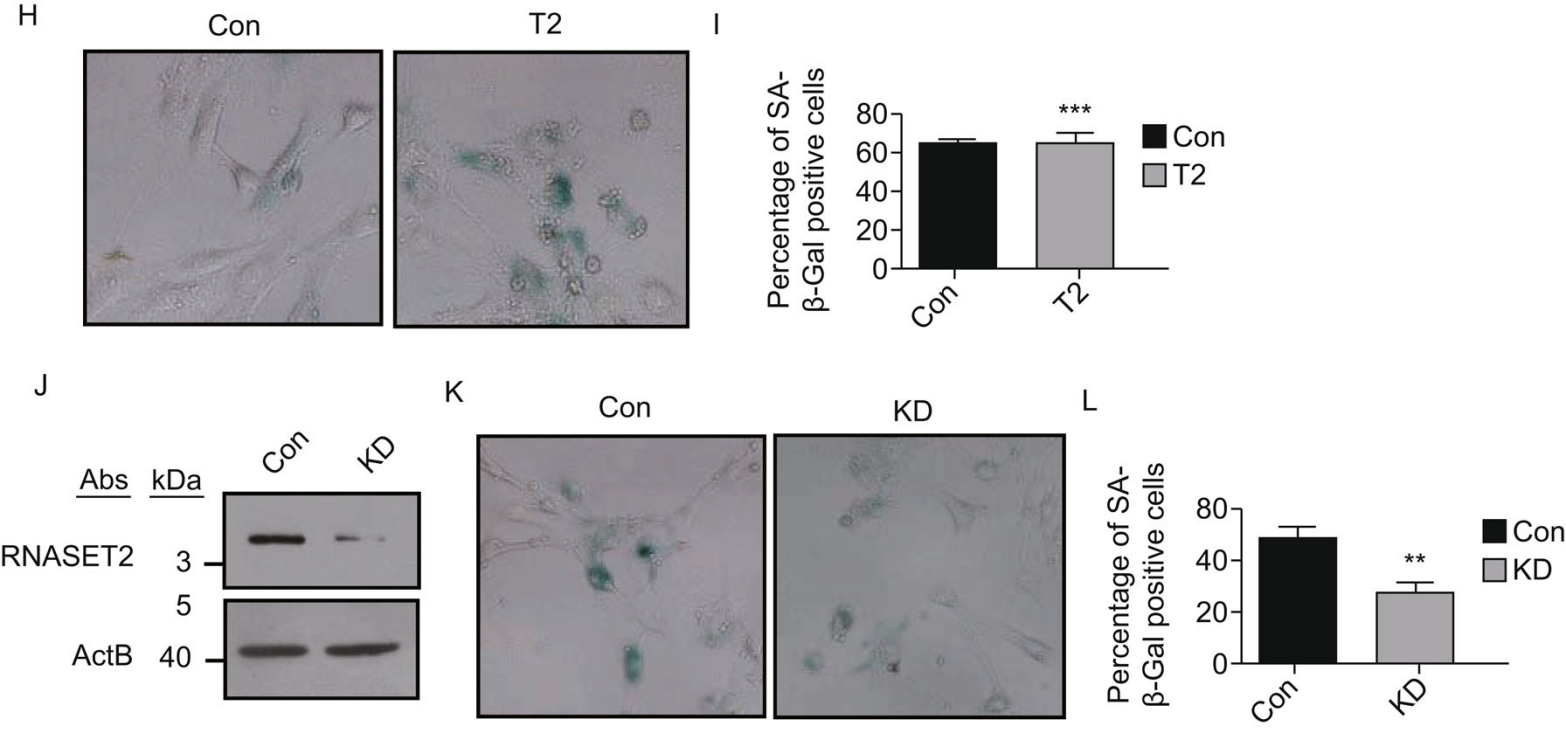
***mTerc-53*accelerates senescence in both wild-type and*terc***^**−/−**^**MEF cells**. (A) Wild-type MEF cells (WT) or *terc*^−/−^ (KO) MEF cells were infected with adenovirus for expression of yeast *CYC1* RNA (CYC1), full length *mTerc* (mTerc-full), *mTerc-53* (mTerc-53) or *mTerc-53r* (mTerc-53r), and stained for SA-β-gal on day 9. (B) Quantification of the β-gal positive cells in panel (A). 10 fields and about 500 cells were counted for each line. (C) Immunoblots of the MEF cell lysates with *mTerc-53* or *mTerc-53r* overexpression. (D) Quantification of the relative p16 level in panel (C) (*n* = 3). (E) Northern blots of cytosolic *mTerc-53* in wild type MEFs with (mTerc-53r) or without (con) *mTerc-53r* overexpression. (F) Comparison of the telomere length in wild-type MEF cells (WT) or *terc*^−**/**−^ (KO) MEF cells infected with adenovirus for expression of yeast *CYC1* RNA (CYC1), full length *mTerc* (mTerc-full), *mTerc-53* (mTerc-53) or *mTerc-53r* (mTerc-53r). (G) Immunoblots of the MEF cell lysates with (con) or without RNASET2 overexpression. (H) SA-β-gal staining of MEF cells with (con) or without RNASET2 (T2) overexpression on day 9. (I) Quantification of the β-gal positive cells in panel (H). (J) Immunoblots of the MEF cell lysates with (con) or without RNASET2 knockdown. (K) SA-β-gal staining of MEF cells with (con) or without RNASET2 knockdown (KD) on day 14. (L) Quantification of the β-gal positive cells in panel (K). Statistical comparisons are performed using unpaired *t*-tests; *P < 0.05, **P < 0.01, ***P < 0.001, ****P < 0.0001. Data are presented as mean ± standard error of the mean (s.e.m.)

To further examine whether *TERC-53* functions by interfering with the functions of full length *TERC*, MEF cells were isolated from second generation *terc*^−/−^ embryos or control wild-type embryos, and infested with adenovirus for the expression of *CYC1*, *mTerc*, *mTerc-53* or *mTerc-53r*. Consistent with the 2BS cell results, *mTerc-53* overexpression accelerated wild-type MEF cell senescence and an increase of p16 protein level was observed in *mTerc-53* overexpressing cells (Fig. 3A–D). *mTerc-53r* had only minor effect on the *mTerc-53* level (Fig. 3E), hence a lack of rescue effect on senescence. More importantly, *mTerc-53* also accelerated senescence of *terc*^−/−^ MEFs (Fig. 3A–D). No deceleration of senescence of *terc*^−/−^ MEFs by expression of *mTerc* was observed, possibly due to the fact that *mTERC* deletion only has minor effect on cellular senescence in early generations (Blasco et al., 1997), and that introducing *mTerc* later in proliferation in the wild-type cells actually increased the level of *mTerc-53*, hence the negative effect outweighing the positive effect. Telomere length was examined in the wild-type and *terc*^−/−^ MEF cell lines. As expected, the telomeres in the *terc*^−/−^ MEFs were shorter than the wild-type MEFs, but expression of *CYC1*, *mTerc*, *mTerc-53* or *mTerc-53r* in these second generation MEFs had no significant effect on telomere length (Fig. 3F). These results suggest that m*Terc-53* functions independent of full length *mTerc* and telomerase, and also provide an explanation for the lack of strong biological phenotypes in early generations of *terc*^−/−^ mice (Blasco et al., 1997; Rudolph et al., 1999; Jaskelioff et al., 2011), as deletion of *mTERC* also eliminates the generally pro-senescence *mTerc-53*.

It has been shown previously that mitochondrion-localized RNASET2 is involved in *TERC* processing, and that overexpression of RNASET2 leads to an increase of cytosolic *TERC-53* level (Cheng et al., 2018). With the involvement of *TERC-53* in cellular senescence established, we examined whether RNASET2 has a similar effect on cellular senescence as *TERC-53* does, by overexpressing or knocking down the protein in MEF cells (Fig. 3G–L). An acceleration of cellular senescence was observed with RNASET2 overexpression and a deceleration of senescence was observed with RNASET2 knockdown (Fig. 3G–L), consistent with a previous report that in human RNASET2 functions as a senescence inducing and tumor suppressor protein (Acquati et al., 2001), and providing a linkage between the processing and function of *TERC-53*.

### *mTerc-53* is involved in cognition decline in 10 months old mouse hippocampus

**Figure 4:**
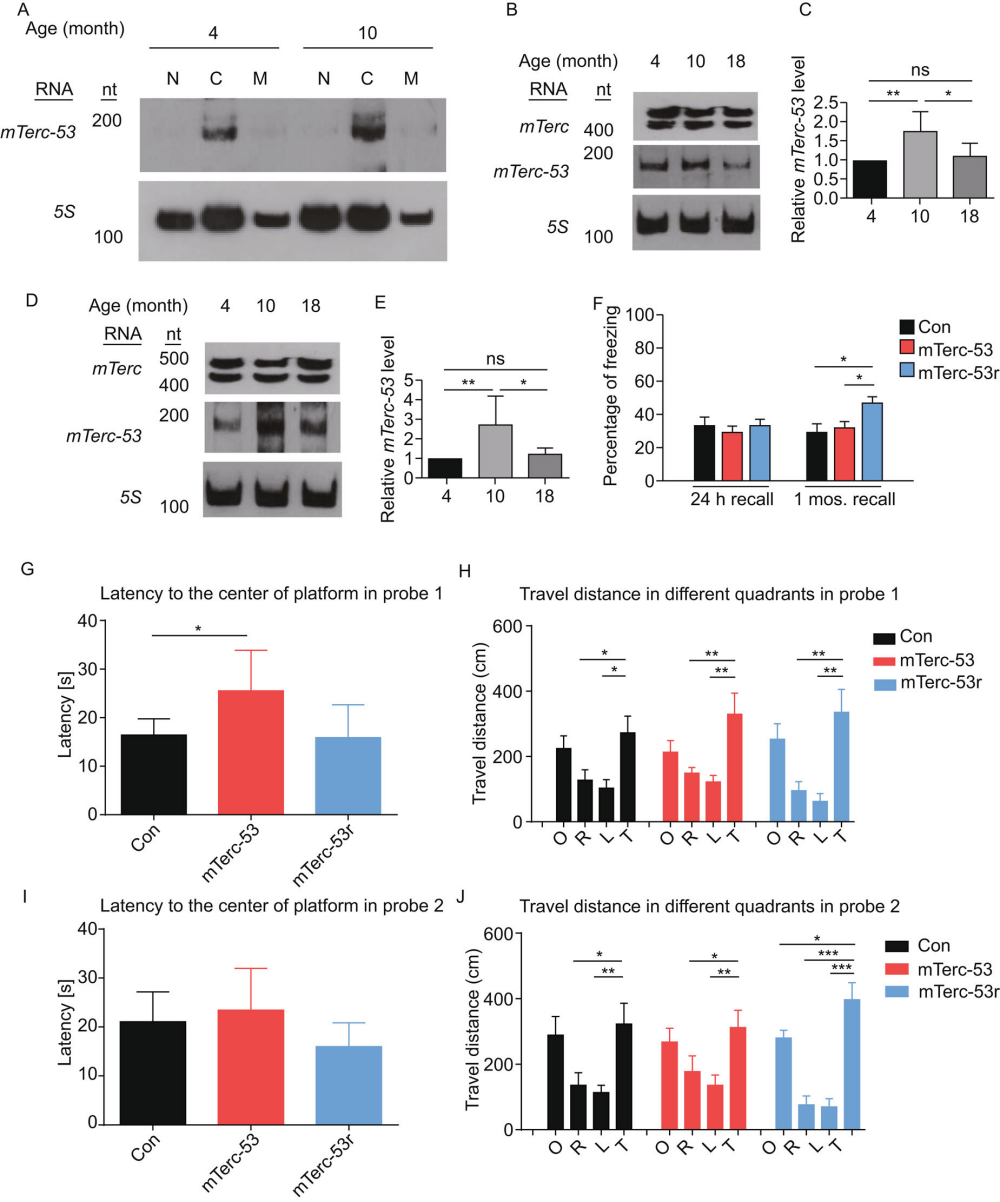
**m*Terc-53*regulates cognition decline in ageing hippocampus**. (A) Northern blots of *mTerc-53* in different cellular fractions (N: nucleus, C: cytosol, M: mitochondria) isolated from the brains of 4 months old mice and those of 10 months old. (B) Northern blots of cytosolic *mTerc-53* in the brains of 4 months, 10 months and 18 months old mice. (C) Relative cytosolic *mTerc-53* levels in the brains of the three groups of mice (*n* = 8 animals). (D) Northern blots of cytosolic *mTerc-53* in the livers of 4 months, 10 months and 18 months old mice. (E) Relative cytosolic *mTerc-53* levels in the livers of the three groups of mice (*n* = 8 animals). (F) Contextual fear memory acquisition of control mice (*n* = 20) or mice overexpressing *mTerc-53* (*n* = 20) or *mTerc-53r* (*n* = 20) in the hippocampus 24 h (24 h recall) or 1 month (1 mos. recall) after training. (G–J) Morris water maze performance of control mice (*n* = 12) and mice overexpressing *mTerc-53* (*n* = 10) or *mTerc-53r* (*n* = 10) in the hippocampus, Panel (G): latency in probe 1; Panel (H): travel distance in probe 1; Panel (I): latency in probe 2; Panel (J): travel distance in probe 2. O, R, L and T stand for Opposite, Right, Left and Target quadrant. The Statistical comparisons are performed using unpaired *t*-tests; *P < 0.05, **P < 0.01, ***P < 0.001, ****P < 0.0001. Data are presented as mean ± standard error of the mean (s.e.m.)

We proceeded to study whether there is a physiological connection of the *TERC-53* function. Cytosolic *mTerc-53* was observed in all mouse tissues examined (Fig. S2A). In cultured neuronal N2a cells, *mTerc-53* also localizes mainly in the cytosol and the level is significantly higher than in MEF TM6 cells (Fig. S2B–D). Moreover, N2a mitochondria import *mTerc* more efficiently, even though PNPASE expression is not upregulated (Fig. S2E–G). Instead, higher TIM23 level was observed in N2a cells, suggesting a correlation between mitochondrial functional states and the processing and trafficking of *mTerc* (Fig. S2G and S2H). Next, the RNA level was examined in different age groups of mice. Majority of *mTerc-53* localizes in the cytosol of the brain cells, consistent with the results in the cultured cells (Fig. 4A). A significant increase of the cytosolic *mTerc-53* level was observed in the brains as well as the livers of the 10 months old mice compared to the 4 months old; but in the 18 months old mice, the level drops again to that of the 4 months old (Fig. 4A–E), which seems consistent with the responses of cytosolic *hTERC-53* levels to different degrees of mitochondrial stresses (Cheng et al., 2018). The PNPASE level remains constant in all three groups, but an ageing-related mitochondrial protein BAP37 increases significantly in the 18 months old mice (Fig. S3A and S3B) (Coates et al., 1997), indicating changes of mitochondrial functional states as mice age. Transcription inhibitor triptolide treatment did not alter the pattern of *mTerc-53* levels in the three groups (Fig. S3C), suggesting no clear change of the degradation patterns of the RNAs during this ageing period. As expected, the telomere length in the brain showed no significant difference among the three age groups (Fig. S3D). Taken together, these data suggest that *mTerc-53* may play a regulatory role in organismal ageing.

To further examine *mTerc-*53’s physiological functions, we decided to use mouse hippocampus as a model and use adenoviral gene delivery, to avoid interference of telomerase activity during development and maturation (Fig. S4A). Contextual fear conditioning (CFC) (Fig. S5A) and spatial learning (Fig. S6A) were performed in separate cohorts. For CFC, mice (10 months old) were trained four weeks after adenovirus injection. Three groups exhibited comparable acquisition of contextual fear memory in single trial 24 h after training, but significantly higher percentage of freezing was observed in mTerc-53r group mice in 1-month recall compared to the control group and mTerc-53 group (Fig. 4F). No difference of the exploratory area and travel distance was observed among the three groups, suggesting no changes in mobility (Fig. S5B and S5C).

For spatial learning, mice were trained 8 weeks after injection. mTerc-53 group exhibited a slower learning curve to locate the hidden platform across 10 training days (Fig. S6A and S6B). The latency of mTerc-53 group to locate the platform in probe 1 was also significantly longer than the control group, suggesting a cognitive decline (Figs. 4G and S7). Each group preferred the Target quadrant to Left and Right quadrants, and the frequency of reaching the platform by each group was about equal, but the ratio of travel distance in quadrant T to that in quadrant L was significantly higher in mTer-53r group (Figs. 4H, S6C–E and S7). After three days’ rest, the ratio of travel distance in quadrant T to that in quadrant L remained significantly higher in mTer-53r group and the frequency of reaching the platform by mTer-53r group also appeared higher, suggesting a better retention of memory (Fig. 4I, 4J, S6C–E and S7). In summary, overexpression of *mTerc-53r* slowed down the cognitive decline in the 10 months mice, while *mTerc-53* accelerated the decline of learning ability in the 10 months mice. The relatively milder effect of *mTerc-53* could possibly be due to induction of the expression of *mTerc-53r* in the hippocampus as a potential compensatory mechanism (Fig. S4B). No effect by *mTerc-53* or *mTerc-53r* was observed in 18 months mice, indicating an early intervention is required (Fig. S6F).

### *mTerc-53* affects cell proliferation and stem cell number in 10 months old hippocampi

**Figure 5:**
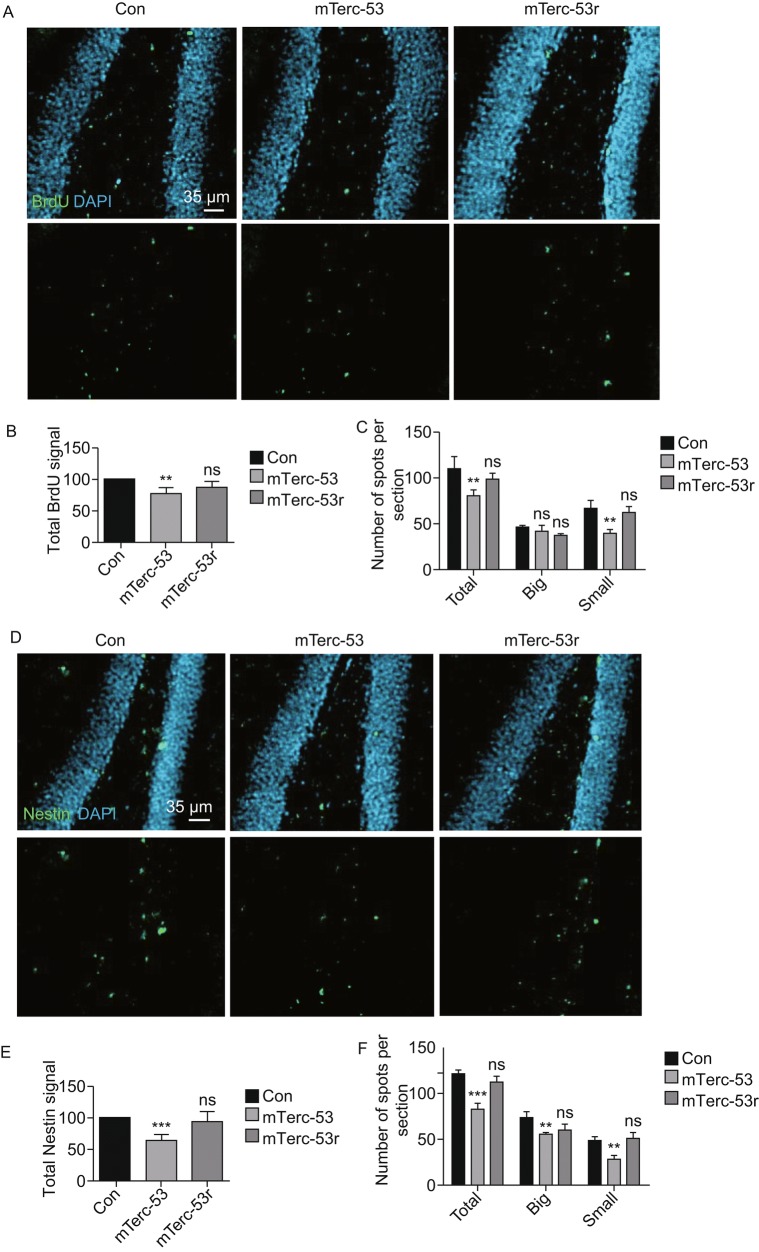
**m*Terc-53*regulates cell proliferation and stem cell number in ageing hippocampi**. (A) Brdu incorporation assay on the three groups of mice. Upper panels are overlays of BrdU signals and DAPI signals. Bottom panels are BrdU images. (B) Quantification of the total BrdU signal in the hippocampus in panel (A). (C) The number of BrdU positive spots in the hippocampi of the three groups in panel (A). 24 slices were counted for each group (*n* = 6). (D) Nestin (Stem cell marker) immunostaining of the hippocampi of the tree groups. Upper panels are overlays of Nestin signals and DAPI signals. Bottom panels are Nestin images. (E and F) Nestin (Stem cell marker) immunostaining of the hippocampi of the tree groups, Panel (E): total signal; Panel (F): number of foci. 24 slices were counted for each group. the Statistical comparisons are performed using unpaired *t*-tests; *P < 0.05, **P < 0.01, ***P < 0.001, ****P < 0.0001. Data are presented as mean ± standard error of the mean (s.e.m.)

To understand the role of *TERC-53* in cognition decline in 10 months old hippocampi, immunohistochemistry was used to evaluate the hippocampi of the mice overexpressing *mTerc-53* or *mTerc-53r* and the control mice. Although no clear difference was observed with FOX3/NeuN (Neuron marker) staining, or S100β (Glial marker) staining, differences were observed in both BrdU incorporation (cell proliferation) and Nestin (stem cell marker) staining (Figs. 5 and S8). In hippocampi overexpressing *mTerc-53*, both the total intensity of the signal and the number of the BrdU- and Nestin-positive spots decreased significantly, compared to the control hippocampi or those overexpressing *mTerc-53r* (Figs. 5 and S8), suggesting that a reduction of cell proliferation and exhaustion of stem cells could be the reason of cognition decline in the *mTerc-53* overexpressing mice. No clear different was observed with COX/SDH (mitochondrial activity) labeling among the three groups, consistent with the previous finding that *TERC-53* functions downstream of mitochondria and has little effect on mitochondrial functions (Cheng et al., 2018).

### Changes of *TERC-53* level affect nuclear gene expression

**Figure 6:**
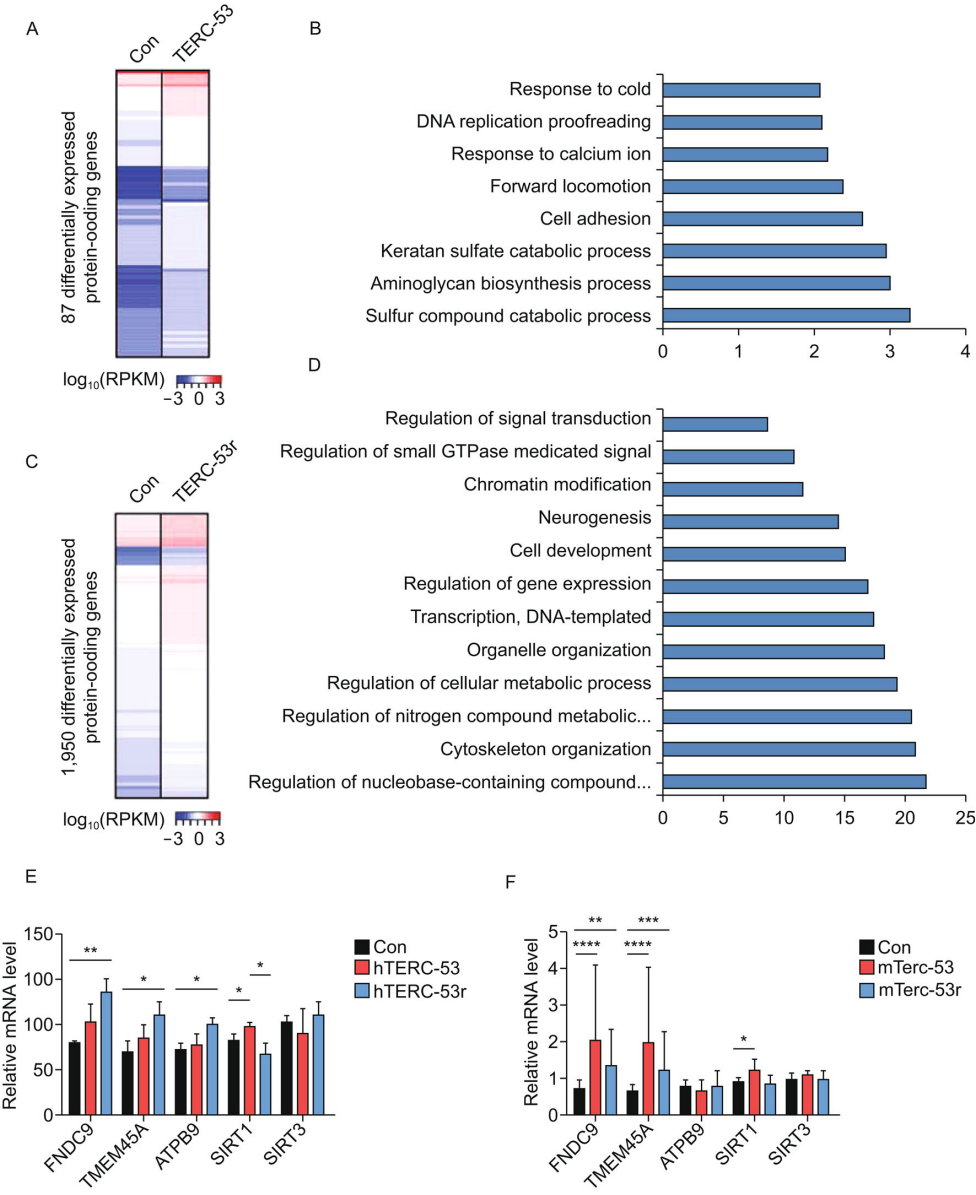
**Exogenous expression of*TERC-53*or*TERC-53r*affects nuclear gene expression**. (A) Differentially expressed (DE) protein-coding genes between HEK cells overexpressing *hTERC-53* and the cells harboring the empty vector. (B) Gene ontology (GO) of the DE protein-coding genes between HEK cells overexpressing *hTERC-53* and the cells harboring the empty vector. (C) DE protein-coding genes between HEK cells overexpressing *hTERC-53r* and the cells harboring the empty vector. (D) GO of the DE protein-coding genes between HEK cells overexpressing *hTERC-53r* and the cells harboring the empty vector. (E) qRT-PCR of *FNDC9*, *TMEM45A*, *ATPB9*, *SIRT1* and *SIRT3* in control, *hTERC-53* or *hTERC-53r* overexpressing HEK cells (*n* = 3). (F) qRT-PCR of *FNDC9*, *TMEM45A*, *ATPB9*, *SIRT1* and *SIRT3* in control, *mTerc-53* or *mTerc-53r* overexpressing hippocampus (n = 8 for each group). Statistical comparisons are performed using unpaired *t*-tests; *P < 0.05, **P < 0.01, ***P < 0.001, ****P < 0.0001. For mouse *FNDC9* and *TMEM45A* qPCR results, F-test was used. Data are presented as mean ± standard error of the mean (s.e.m.)

To gain more mechanistic insight into the function of *TERC-*53, its effect on nuclear gene expression was examined. Total RNA were harvested from HEK293 cells overexpressing *hTERC-53*, *hTERC53r* or the control cells and analyzed using RNAseq. 87 differentially expressed protein coding genes with GFOLD >1 or <−1 were identified between *hTERC-53* overexpressing and control cells, and 1,950 differentially expressed protein coding genes were identified between *hTERC-53r* overexpressing and control cells (Fig. 6A–D and Tables S1–4). qRT-PCR and RNAseq showed generally consistent changes (Fig. 6E). More importantly, qRT-PCR results of HEK293 RNA were also consistent with those of mouse hippocampus RNA (Fig. 6E and 6F). Gene ontology showed majority of differentially expressed genes were upregulated in *TERC-53r* overexpressing cells and mainly participate in several biological processes such as organelle organization, metabolism and neurogenesis, while the differentially expressed genes in *TERC-53* overexpressing cells mainly participate in catabolic processes and stress responsive pathways, such as response to cold and calcium ion (Fig. 6B and 6D), suggesting that *TERC-53* plays a regulatory instead of a causative role in senescence and ageing.

### *TERC-53* regulates GAPDH nuclear translocalization

**Figure 7:**
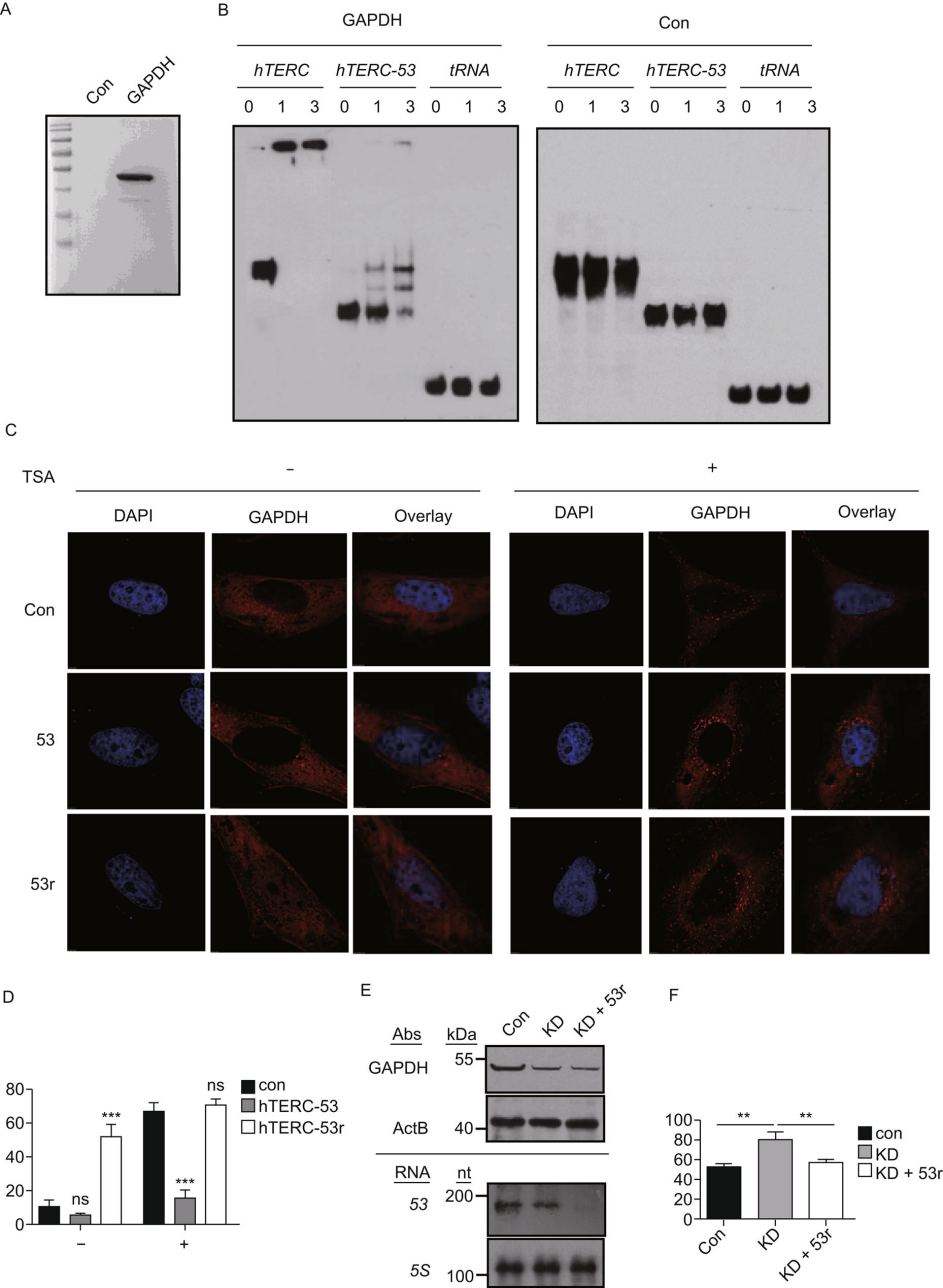
**Exogenous expression of*TERC-53*or*TERC-53r*affects GAPDH nuclear translocalization**. (A) Coomassie staining of the purified GAPDH. (B) Gel shift assay of purified GAPDH with the indicated RNA. (C) GAPDH localization (red) in HEK293 cells overexpressing the indicated RNA, with or without TSA treatment. (D) Quantification of the number of cells with nuclear GAPDH localization in Panel (C). (E) Top panel: immunoblots of HEK293 cells with GAPDH knockdown (KD) or GAPDH knockdown combined with *hTERC-53r* expression (KD + 53r); bottom panel: northern blots of cytosolic *hTERC-53* (*53*) and *5S* rRNA. (F) Percentage of SA-β-gal positive cells after H_2_O_2_ treatment and 3 days’ recovery

*TERC* has been shown to interact with GAPDH in the nucleus, an important component of a gene transcriptosome, and nuclear translocalization is a key step for GAPDH to participate in the gene expression regulation (Sawa et al., 1997; Hara et al., 2005; Sen et al., 2008; Nicholls et al., 2012). Translocalization of GAPDH into the nucleus is induced by exposure of cells to environmental stressors such as starvation or treatment with Histone deacetylase inhibitor TSA (Trichostatin A), and plays an important role in DNA repair, autophagy and cell death (Chuang and Ishitani, 1996; Nagy et al., 2000; Azam et al., 2008; Chang et al., 2015). To examine whether *TERC-53* could interact with the cytosolic pool of GAPDH and interfere with its nuclear translocalization or functions, GAPDH was purified and incubated with in vitro synthesized *hTERC-53*. A specific binding of GAPDH to *hTERC-53* and *hTERC* but not control tRNA was observed (Fig. 7A and 7B). When TSA (Trichostatin A) was used to induce GAPDH nuclear translocation, in HEK 239 cells overexpressing *hTERC-53*, much less GAPDH signal was observed in the nucleus, compared to the control cells or the cells overexpressing *hTERC-53r* (Fig. 7C and 7D). Interestingly, even without TSA treatment, GAPDH accumulated in the nucleus of the cells overexpressing *hTERC-53r*, suggesting that *hTERC-53* functions by interfering with the nuclear translocation of GAPDH, and subsequently affecting nuclear gene expression (Fig. 7C and 7D). It has been shown previously that GAPDH depletion accelerates cellular senescence of a cancer line (Phadke et al., 2011). To examine whether *TERC-53* plays a role in the process, *hTERC-53r* was expressed in the GAPDH knockdown cell line (Fig. 7E). As expected, GAPDH knockdown led to an increase of the senescence marker but depletion of *hTERC-53* by expression of the antisense RNA partially reversed the effect (Fig. 7F), suggesting interfering with GAPDH nuclear translocalization as a mechanism of *TERC-53*’s function in cellular senescence.

## Discussion

Mitochondria import a variety of cytosolic RNAs, but the functions of the majority of these RNAs in mitochondria are unclear. We have previously shown that the RNA component of Telomerase is imported into mitochondria, processed to a shorter form *TERC-53*, and then exported back to the cytosol (Cheng et al., 2018). The cytosolic *TERC-53* level acts as an indicator of mitochondrial functions, but has no direct effect on mitochondria (Cheng et al., 2018). Our results in this report show that cytosolic *TERC-53* regulates cellular senescence and is involved in cognition decline in 10 months old mouse hippocampi through a telomerase independent mechanism. We also provide a possible mechanism on how cytosolic *TERC-53* regulates nuclear gene expression. We have shown here a non-coding RNA functioning as a signaling molecule for regulation of nuclear gene expression, which appears to play a regulatory role in senescence.

Ageing in mammals is characterized by gradual loss of physiological functions. Mutations in mitochondrial DNA and mitochondrial dysfunctions have been established as one of the major factors in ageing (Lopez-Otin et al., 2013). Studies in mammals show an important correlation between nuclear gene expression and mitochondrial function changes during ageing (Lu et al., 2004; Bishop et al., 2010). How some mitochondrial dysfunction elicits certain nuclear gene expression change and how the specificity is achieved, however, is not clear. Our findings suggest that *TERC-53* may function as a specific signaling molecule that bridges mitochondria and the nucleus. As the effect of mitochondrial dysfunctions on ageing is not simply causative, so is the effect of *TERC-53*. Modest reduction of mitochondrial function has been shown to activate a compensatory mechanism that increases stress resistance (Schulz and Haynes, 2015). Similarly, we have observed an induction of stress response pathways by *TERC-53* overexpression. Cytosolic *TERC-53* level responds differently to different levels of mitochondrial stress, it increased initially but then decreased as the stress increases. Similar changes were observed in mice as they age. These findings paint a complex picture of regulation networks during ageing in mammals. There is constant deterioration of the overall structures, as shown by accumulation of mutations in mitochondrial DNA and nuclear DNA, oxidation of the membranes, and so on; but there are also many coping mechanisms (Bishop et al., 2010). Noncoding RNAs may play a bigger role in ageing in mammals than expected.

One of the most important finding of this study is that *mTerc-53* accelerates cellular senescence of second generation *terc*^−/−^ MEFs. *TERC* is the essential RNA component of telomerase, and the activity of telomerase plays a big role in cellular senescence (Gall, 1990; Bernardes de Jesus and Blasco, 2013). That exogenous expression of cytosolic *TERC-53* has a similar impact on senescence of *terc*^−/−^ cells as wild-type cells suggests *TERC-53* does not function by interfering with telomerase activity. More importantly, it also provides an explanation for the lack of strong biological phenotypes in early generations of *terc*^−/−^ mice. Full length *mTerc*, as an essential component of telomerase, has a pro-longevity function, while its processed form *mTerc-53* is generally pro-senescence. Deletion of *mTerc* essentially eliminates the pro-senescence *mTerc-53.* The effect of *TERC-53* in cognition decline seems to be caused by its effect on cell proliferation, at least in mouse brains, which is consistent with its function on cellular senescence. Although we have evidence showing *TERC-53* regulates nuclear gene expression and the nuclear translocation of GAPDH, a key component of a transcriptosome, the molecular mechanism of *TERC-53*’s function in general and in cellular senescence remains to be fully elucidated.

## Methods

### Subcellular fractionation

Mitochondria were isolated as previously described (Hachiya et al., 1993), except that mitoprep buffer (0.225 mol/L mannitol, 0.075 mol/L sucrose and 20 mmol/L HEPES pH 7.4) was used as the homogenization buffer and wash buffer. For cytosol isolation, post mitochondrial supernatant was first spun at 21,000 ×*g* for 10 min at 4 °C, and the supernatant was again spun at 100,000 ×*g* for 30 min at 4 °C. Nuclei and unbroken cells from mitochondrion isolation were pelleted at 800 ×*g* at 4 °C. The pellet was resuspended in the mitoprep buffer, homogenized again on ice, and pelleted at 800 ×*g* at 4 °C. The last step was repeated twice, before the pellet was resuspended in lysis buffer (10 mmol/L HEPES pH 7.5, 10 mmol/L KCl, 0.1 mmol/L EDTA, 1 mmol/L DTT, 0.5% Nonidet-40). The sample was kept on ice for 10 min, and nuclei were pelleted at 12,000 ×*g* for 10 min at 4 °C.

### Isolation of total and mitochondrial RNA

100 μg Mitochondria were treated with digitonin (90 μg/mg mitochondria) and 300 U micrococcal nuclease (Thermo) in 200 μL mitoprep buffer with 1 mmol/L CaCl_2_ for 25 min at room temperature. The reaction was stopped by addition of 5 mmol/L EDTA. Mitochondria were collected and solubilized in 100 μL SDS buffer (100 mmol/L NaCl, 1% SDS, 20 mmol/L Tris pH 7.4) with 10 μg/mL proteinase K and 5 mmol/L EDTA at 50 °C for 5 min. RNA was purified using 400 μL TRIzol reagent (Invitrogen), and treated with 500 U RNase-free DNase I (Thermo) in 50 μL buffer for 25 min at 37 °C. DNase I was inactivated with the addition of 5 mmol/L EDTA and incubation at 70 °C for 10 min. RNA was then purified with TRIzol. RNAseq samples and qRT-PCR samples were prepared the same way.

### RT-PCR

RT-PCR was performed using the AccessQuick RT-PCR kit (Promega). For detection of *hTERC*, hTERC-111-f 5′-ACTTTCAGCGGGCGGAAAAGCCTCG-3′ and hTERC-198-r 5′-GCGAACGGGCCAGCAGCTGACATTT-3′ were used as primers, and for detection of *GAPDH*, GAPDH-f 5′-GAGTACGTCGTGGAGTC-3′ and GAPDH-r 5′-GGTCCACCACCCTGTTG-3′ were used. For detection of *COX2*, cox2-f 5′-CGGCCGCACCGGTGCACATGCAGCGC-3′ and cox2-r 5′-CGCGGATCCCTATGGTAAATACGGGC-3′ were used. For detection of *CYC1*, cyc1-f 5′-ATGACTGAATTCAAGGCCGGTTCTG-3′ and cyc1-247-r 5′-TCAACCCACCAAAGGCCATC-3′ were used. Phusion High-Fidelity DNA Polymerase was used for PCR of *TERC* and *TERC-53.*

### *In vitro* transcription

RNA was synthesized using MEGAscript SP6 Kit (Ambion). For radiolabeled RNA synthesis, [^32^P]-UTP was incorporated. For biotin-labeled RNA synthesis, biotin RNA labeling mix (Roche) was used. RNA was purified using TRIzol reagent.

### Plasmids

To make the construct pQsuper-hTERC-53 for HEK293 transfection, full length *hTERC* was first RT-PCR amplified from HEK293 total RNA with the primer pair: (hTERC-f) 5′-GGGTTGCGGAGGGTGGG-3′ and (hTERC-r) 5′-GCATGTGTGAGCCGAGTCCT-3′. *hTERC-53* was then PCR amplified from full length *hTERC* with the primer pair: (Bgl2-hTERC-53-f) 5′-GAAGATCTTAACTGAGAAGGGCGTAG-3′ and (Xhol1-hTERC-247-r) 5′-CCGCTCGAGTGCGGGGTTCGGGGGCT-3′. The PCR product was digested with *Bgl*II and *Xho*I, and inserted into pQsuper vector. The pQsuper-hTERC-53r antisense construct and pQsuper-hTERCΔ1-63 were generated with the same method using the primer pair: 5′-CCGCTCGAGTAACTGAGAAGGGCGTAG-3′ and 5′-GAAGATCTTGCGGGGTTCGGGGGCT-3′ and the primer pair: 5′-GAAGATCTGGCGTAGGCGCCGTG-3′ and 5′-CCGCTCGAGGCATGTGTGAGCCGAGTCCT-3′. For 2BS cell transfection, pMSCV-hH1 vector was made. *hH1* promoter fragment was cut from pQsuper vector with *Bam*HI and *Xho*lI and inserted into pMSCV-puro vector digested with *Bgl*II and *Xho*lI to generate pMSCV-hH1. To make the construct pMSCV-hH1-cyc1, *hH1-CYC1* fragment was cut from pQsuper-cyc1 and inserted into pMSCV-puro vector. With the same method, pMSCV-hH1-hTERC-full, pMSCV-hH1-hTERC-53 and pMSCV-hH1-hTERC-53r were also constructed. For mouse cell transfection, *mTERC*, *mTERC-53*, *mTERC53r* were PCR amplified using primers: 5′-GAAGATCTACCTAACCCTGATTTTCATTAGCTGTGG-3′ and 5′-CCGCTCGAGGGTTGTGAGAACCGAGTTCC-3′; 5′-GAAGATCTAGCTCCAGGTTCGCCGGGA-3′ and 5′-CCGCTCGAGGTCCTCGCGGCGCTCGC-3′; 5′-GAAGATCTGTCCTCGCGGCGCTCGC-3′ and 5′-CCGCTCGAGAGCTCCAGGTTCGCCGGGA-3′. The PCR products were digested with *Bgl*II and *Xho*I, and inserted into pQsuper vector. For bacterial expression of GAPDH, Pet28a-GAPDH-flag-his was generated using primers: 5′-CGCGGATCCATGGGGAAGGTGAAG-3′ and 5′-CCGCTCGAGCTCCTTGGAGGCCATG-3′, and BamHI and XhoI insertion. For colocalization study, PQCXIP-GAPDH-mCherry was generated using primers 5′-AAAACCGGTATGGGGAAGGTGAAG-3′ and 5′-CGCGGATCCACTCCTTGGAGGCCATG-3′, and *Age*I and *Bam*HI insertion.

### Cell culture and transfection

HEK293 cells, TM6 cells (Wang et al., 2010), 2BS cells (Li et al., 2011), MEF cells and N2a cells were cultured in DMEM supplemented with 10% fetal bovine serum. All cell lines used were tested for mycoplasma contamination. To generate HEK293 stable cell lines, HEK293T cells were transfected with VSVg and Hit60 packaging vectors and the vector of interest using TurboFect (Thermo). Harvested retroviruses were used to infect HEK293 cells, followed by selection with 5 μg/mL puromycin. To generate 2BS stable cell lines, phoenix cells were transfected with the vector of interest using CaCl_2_ transfection protocol. Harvested retroviruses were used to infect 2BS cells at 20 PDs, followed by selection with 0.5 μg/mL puromycin. RNASET2 overexpression and knockdown was performed as previously described (Liu et al., 2017).

### Western blot

Cells were washed twice in 1× PBS, pH 7.4, and lysed in buffer A (10 mmol/L HEPES, pH 7.9, 10 mmol/L KCl, 1.5 mmol/L MgCl_2_ and 0.5% NP-40). Mitochondria were lysed directly in 1× SDS loading buffer. Protein lysates (50 μg) were resolved by SDS-PAGE, transferred to nitrocellulose membranes, incubated for 1 h with 5% milk TBS-T and overnight with primary antibodies in 5% BSA at 4 °C or for 1–2 h at room temperature. Antibodies included anti-PNPASE (1:5,000) (Chen et al., 2006), anti-TIM23 (1:1000) (Abgent), anti-Mortalin (1:10,000) (Sigma-Aldrich), anti-Creb (1:1000) (Abcam), anti-ActB (1:2,000) (ABClonal), anti-Acetylated lysine (1:1000) (Cell Signaling Technology), anti-TERT (1:1000) (Abcam), anti-p-AMPK (Thr172) (1:1000) (Cell Signaling Technology), anti-SIRT1 (1:500) (Santa Cruz Biotechnology), anti-SIRT6 (1:1000) (Cell Signaling Technology), anti-SIRT3 (Cell Signaling Technology), anti-p-NF-kB(Ser536) (1:1000) (Cell Signaling Technology), anti-p16 (1:1000) (Bioworld) and anti-β-Tubulin (1:2000) (Abcam).

### Northern blots

RNA was extracted from the nucleus, the cytosol or mitochondria using TRIzol reagent. 2 μg nuclear RNA, 5 μg cytosolic RNA and 0.5 μg mitochondrial RNA were loaded onto 6% polyacrylamide-8 mol/L urea gels, transferred to Amersham Hybond N+ membrane (GE) and hybridized with biotin-labeled RNA probes. For *hTERC-53* detection, biotin-labeled *hTERC-53* antisense RNA probe was used. For *5S* rRNA, biotin-labeled full length *5S* antisense RNA probe was used. Experiments were performed using North2South Hybridization and Detection Kit (Thermo).

### Senescence-associated beta-galactosidase staining

Senescence Cell Histochemical Staining Kit (Sigma) was used. Cells were washed twice in PBS and then fixed to plates using 1× Fixation Buffer for 6–7 min. After fixation, the cells were washed with PBS 3 times and incubated overnight at 37 °C without CO_2_ in freshly prepared Staining Mixture. For induction of HEK293 cellular senescence, cells were treated with 400 μmol/L of H_2_O_2_ for 24 h, and then grown in normal medium without H_2_O_2_ for 3 days before beta-Galactosidase Staining.

### Telomerase activity test

Telomerase activity test was performed using TRAPEZE Telomerase Detection Kit (Millopore). Cells were lysed in CHAPS lysis buffer containing 200 units/mL RNase inhibitor. 1.5 μg cell extract were used per assay. The PCR products were analyzed on a 12.5% non-denaturing PAGE and detected by SYBR Green staining.

### Gel shift assay

1–2 ng biotin labeled RNA was incubated with 0, 0.5, or 1.5 μg purified GAPDH in 10 μL binding buffer (150 mmol/L KCl, 25 mmol/L Tris pH 7.4, 5 mmol/L EDTA, 0.5 mmol/L DTT, 5% glycerol, 100 U/mL RNase Inhibitor (Thermo)) at 30 °C for 20 min. The samples were then kept on ice for 5 min; 10 μL of 2× RNA loading buffer was added to the mixture, and the samples were subjected to TBE gel electrophoresis.

### Fluorescent microscopy

Hela cell lines stably expressing *hTERC-53*, or *hTERC-53R*, or harboring the empty vector were transiently transfected with PQCXIP-GAPDH-mCherry for 2 days. The cells were treated with 100 nmol/L TSA (ApexBio) or DMSO for 24 h, stained with DAPI, and then analyzed with a Zeiss confocal microscopy.

### MEFs culture

MEFs were isolated as previously described (Jozefczuk et al., 2012) and cultured in DMEM containing 10% FBS and non-essential amino acids. On day 3, the cells were infected with adenovirus. After two days, the cells were treated with 2 μg/mL puromycine for 24 h. β-Gal assay was performed on day 9 with Senescence Cell Histochemical Staining kit (Sigma).

### Telomere length assay

Genomic DNA was extracted from tissues and cells with Multisource Genomic DNA miniprep kit (Axygen). For measuring telomere length, quantitative real-time PCR methods were used as previously described (Cawthon, 2009; Min et al., 2018).

### Animal studies

All animal studies were performed in strict accordance with guidelines of Chinese Association for Laboratory Animal Science. Protocols were approved by the Animal Care and Use Committee at Tsinghua University.

### Mouse tissue *TERC-53* analysis

C57BL/6 male mice were used to analyze the level *mTerc-53* in the young and the old. The young were 4 months old and the old were 10 months old. The mice were divided into pairs randomly. One pair were sacrificed each time and the fresh livers and brains were used to isolated different cellular fractions. RNA was then isolated from these fresh samples and analyzed by Northern blotting. Biotin-labeled *mTerc* 37~204 bp antisense RNA probe was used to detect *mTerc-53*. 5S rRNA was used as internal control. The Northern blot results (*n* = 8 for each group) were analyzed with ImageJ software. For analysis of cytosolic *TERC-53* levels in tissues, cells were isolated by treating tissues with 2 mg/mL Collagenases (Yeasen) (Type II for muscle and Type I for all the others) in regular growth medium before isolation of different cellular fractions.

### Adenoviral injections

*m-TERC53* and *m-TERC53r* expressing vectors were constructed with BLOCK-iT^TM^ U6 RNAi Entry Vector Kit (Invitrogen) according to the supplier’s instruction, and the virus were produced, amplified and purified with BLOCK-iT^TM^ Adenoviral RNAi Expression System (Invitrogen) according to the supplier’s instruction. Mice were maintained under standard housing conditions, and anaesthetized with Isoflurane (3 mL/h for 10 min to induce anaesthetization, 1.2 mL/h to maintain the anaesthetized state). Mice were placed in the stereotaxic apparatus and a small hole was drilled at each bilateral injection location. 0.2 μL virus (0.1 OD) was injected per site using a Hamilton microsyringe (0.02 μL/min) into the dorsal DG using the following coordinates: anterioposterior = − 2.2 mm from bregma; lateral = ± 1.5 mm; ventral = 2.3 mm. The skin incision was closed carefully after adenoviral injection to minimize inflammation. Injection needles were left in place for 5 min before and after injection to ensure right location and even distribution of the virus.

### Behavioral experiments

Age-matched, genotype-matched male B6 mice (middle-aged 10 months) were used for all behavioral experiments. Contextual fear conditioning (CFC) and spatial learning were performed in separate cohorts. For CFC, mice were trained 4 weeks after adenovirus (mTerc-53/mTerc-53r/con) injection. For spatial learning, mice were trained 8 weeks after AV (mTerc-53/mTerc-53r/con) injection.

### Contextual fear conditioning

The contextual fear conditioning (day 0) protocol entailed delivery of a single 2 s footshock of 0.8 mA, 180 s after habituation of the mouse in the training context. The mouse was taken out 10 s after termination of the footshock. Freezing levels were quantified over the 180 s habituation phase prior to the shock. On day 1, animals were exposed to the training context (in which they did not receive a shock) for three minutes to test the 24 h recall. From the end of 24 h recall to the initial of 1 mos. recall, mice were kept in their own home cage respectively. On day 30, animals were exposed to the training context (in which they did not receive a shock) for three minutes again, to test the 1 mos. recall. Conditioning was conducted in TSE multi CFC arena with clear front and back Plexiglas walls, black side walls, and stainless-steel bars as a floor. The arena was lit from above with a light, given back sound by a speaker, ventilated with a fan, and encased by a sound-dampening cubicle. Mouse behavior was recorded by digital video cameras mounted above the conditioning arena. For the training context (designated A throughout), the fan was on, the room light was on level 500, and the back sound was on level 1 as a white noise. Stainless-steel bars were exposed, and 1.5% HAc was used as an olfactory cue. Mice were brought into the training arena in a standard housing cage. Animals were counterbalanced for order of recalls, with the second exposure occurring 29 days following the initial test. TSE multi conditioning system software was used for recording and analyzing freezing behavior.

### Morris water maze tasks

The task was performed with one training phase: acquisition phase (11 days, Q4). A probe trial, in which the mice were released at the center point at the edge of the Opposite quadrant and allowed to swim for 60 s in the absence of the platform, was performed 24 h after the last trial of the acquisition phase (day 10) and twice 3 days after the first probe. The animals’ trajectories were recorded with a video tracking system (EthoVision XT). The apparatus consisted of a white pool 120 cm in diameter and 60 cm deep, filled with water to a depth of 45 cm. Four black shapes were equally spaced on the walls of the room as visible cues. Water temperature was maintained at approximately 23 °C by an automatic thermostatic system. A clear Plexiglas goal platform 9 cm in diameter was positioned ~0.5 cm below the surface of the water (hidden platform), approximately 30 cm from the wall of the tank. Latency and frequency to reach the platform were recorded for training trials, while swim distance in quadrant were calculated for probe trials. 3 trial sessions were performed on days 0–10, with mice released facing the wall in the center point of the edge of each quadrant except Target quadrant, in a pseudorandom order such that no single start location was used in consecutive train (~1 h. interest interval). Mice were allowed to swim for 60 s, and the animals that failed to locate the platform in this time were guided to the platform and allowed to rest for 30 s before being removed from the tank. The latency to reach the platform was averaged for each day. Probe trials were performed on day 11 and day 14, with mice released facing the wall in the center point of the edge of opposite quadrant. Mice were allowed to swim for 60 s before being removed from the tank. Mice were gently dried after each trial in both training and probes.

### Statistical analysis

Statistical analysis was carried out using GraphPad Prism software. Unpaired one-tailed Student’s *t*-tests were used to compare three groups or to compare preference for each quadrant in each group. To compare groups across training days for the Morris water maze, two-way repeated measures ANOVA was used. For contextual fear conditioning, behavioral data were scored by TSE multi conditioning system software. The sample size was chosen based on a previous study (McAvoy et al., 2016) and its power was validated with result analysis. Blinding was applied during the experiment. Randomization was used for selection of animals for each behavioral experiment.

### Immunohistological analysis

Following anesthesia, mice were sacrificed, and heart perfusion with 4% PFA (EMS) was performed. Whole brains were rapidly harvested and post-fixed in 4% PFA at 4 °C for 24 h. The brains were then embedded in 2% agarose. Coronal brain sections (35 μmol/L) were collected from vibrating microtome (Leica Inc. Germany). The slices were dried on cation coated slides at 4 °C overnight and stored without coverslips at −20 °C until ready to use. Slides were then taken out of −20 °C and dried at room temperature for at least 30 min. All sections were antigen-retrieved with citrated acid buffer (pH 6.0) in a microwave oven, permeated with 0.5% Triton for 20 min after washing with PBS, and blocked with 5% GBS in PBST (0.2% Triton) at room temperature for 2 h. The sections were incubated with primary antibodies diluted with 5% FBS in PBST at 4 °C overnight. The next morning, the sections were washed 4 times with PBS, then incubated with secondary antibodies in the dark, washed again, and then mounted with HardSet Mounting Medium (Vectashield) and coverslips after DAPI staining. Slices were scanned with Zeiss Axio Z1 Slide scanner. The primary antibodies include rat anti-BrdU7 (1:500, abclonal), rabbit anti-NeuN (1:500, abclonal), mouse anti-Nestin (1:200, abcam) and rabbit anti-S100β (1:200, abcam). The secondary antibodies include goat anti-mouse IgG Alexa 488 (1:1000, Thermo), goat anti-rabbit IgG Alexa 594 (1:1000, Thermo). For BrdU incorporation, 1 mg/g BrdU in 100 μL of sterile PBS was i.p. injected every 24 h for 1 week before the histological experiments.

### COX and SHD histochemistry

Slides with brain sections were taken out of −20 °C and dried at room temperature for at least 30 min. Reaction buffers were made fresh: for COX staining, 5 mmol/L DAB and 100 μmol/L cytochrome C were added into 0.1 mL PBS (pH 7.0), mixed quickly, before 2 μg of bovine catalase was added; for SDH staining, 1.5 mmol/L NBT, 130 mmol/L sodium succinate, 0.2 mmol/L PMS, and 1 mmol/L sodium azide were added into 0.1 mL PBS (pH 7.0), and mixed quickly in the dark. Slides were incubated with the buffers for 40 min at 37 °C, washed, dehydrated with gradient ethanol, mounted with Hardset Mounting Medium, and scanned with Zeiss Axio Z1 Slide scanner.

### Differentially expression analysis and gene ontology annotations

Tophat (Trapnell et al., 2012) was used to map cleaned RNA sequencing reads to hg19 genome, and GFOLD was then used for differentially expression analysis (Feng et al., 2012). To identify significantly differentially expressed genes, each candidate gene needs to meet two criteria below: i. GFOLD > 1 or GFOLD < −1; ii. *q*-value < 0.05. An R package *topGO* was used for Gene Ontology enrichment analysis. Hypergeometric test was used to measure the significance of GO term enrichments.

## Electronic supplementary material

Below is the link to the electronic supplementary material.

Supplementary material 1 (PDF 1567 kb)

Supplementary material 2 (XLSX 211 kb)
