# Supplementary material for: Mitochondrion-processed TERC regulates senescence without affecting telomerase activities
Source: Protein Cell. 2019 Feb 20;10(9):631–48. doi: 10.1007/s13238-019-0612-5 (PMC6711880; doi:10.1007/s13238-019-0612-5)
Supplement: Supplementary file 1 — Supplementary material 1 (PDF 1567 kb) [file 13238_2019_612_MOESM1_ESM.pdf]

Non-coding RNA *TERC* functions as an ageing-related mitochondrial retrograde signal

Qian Zheng[1*], Peipei Liu[1*], Ge Gao[1], Jinliang Huang[1], Jiapei Yuan[1], Pengfeng Wang[2], Leiming Xie[1], Xinping Lu[1], Fan Di[1], Tanjun Tong[2,3], Jun Chen[2,3], Zhi Lu[1], Jisong Guan[1], Geng Wang[1]

[1]MOE Key laboratory of Bioinformatics, Cell Biology and Development Center, School of Life Sciences, Tsinghua University, Beijing, 100084, China
[2]Peking University Research Center on Aging, Beijing 100191, China
[3]Department of Biochemistry and Molecular Biology, Peking University Health Science Center, Beijing 100191, China
*These authors contributed equally to this work.
Correspondence should be addressed to GW, E-mail: wangeng@biomed.tsinghua.edu.cn

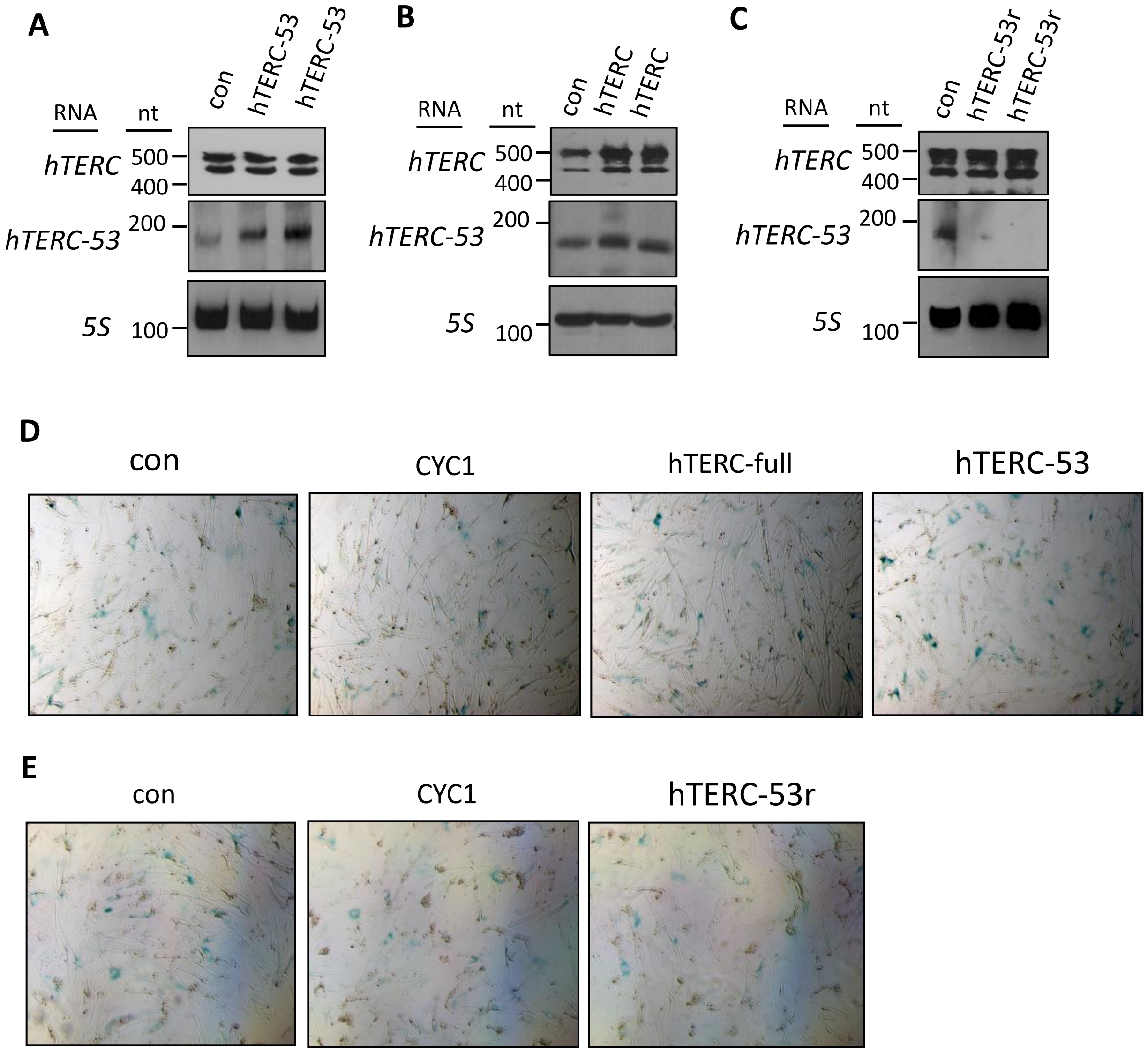

Figure S1. SA-β-gal staining of 2BS cell lines, related to Figure 1.
(A) Northern blots of total *hTERC*, *hTERC-53* and *5S* rRNA in HEK cells (con), or HEK cells overexpressing *hTERC-53* (hTERC-53). (B) Northern blots of total *hTERC-53* and *5S* rRNA in HEK cells (con), or HEK cells overexpressing *hTERC* (hTERC). (C) Northern blots of total *hTERC-53* and *5S* rRNA in HEK cells (con), or HEK cells overexpressing *hTERC-53r* (hTERC-53r). (D) 2BS cell lines generated with the empty vector (con), or the vector expressing yeast *CYC1* RNA (CYC1), full length *hTERC* (hTERC-full) or *hTERC-53* (hTERC-53) were grown to 37 PDs, and then stained for SA-β-gal. (E) 2BS cell lines generated with the empty vector (con), or the vector expressing yeast *CYC1* RNA (CYC1) or anti-sense *hTERC-53* (hTERC-53r) were grown to 43 PDs and stained for SA-β-gal.

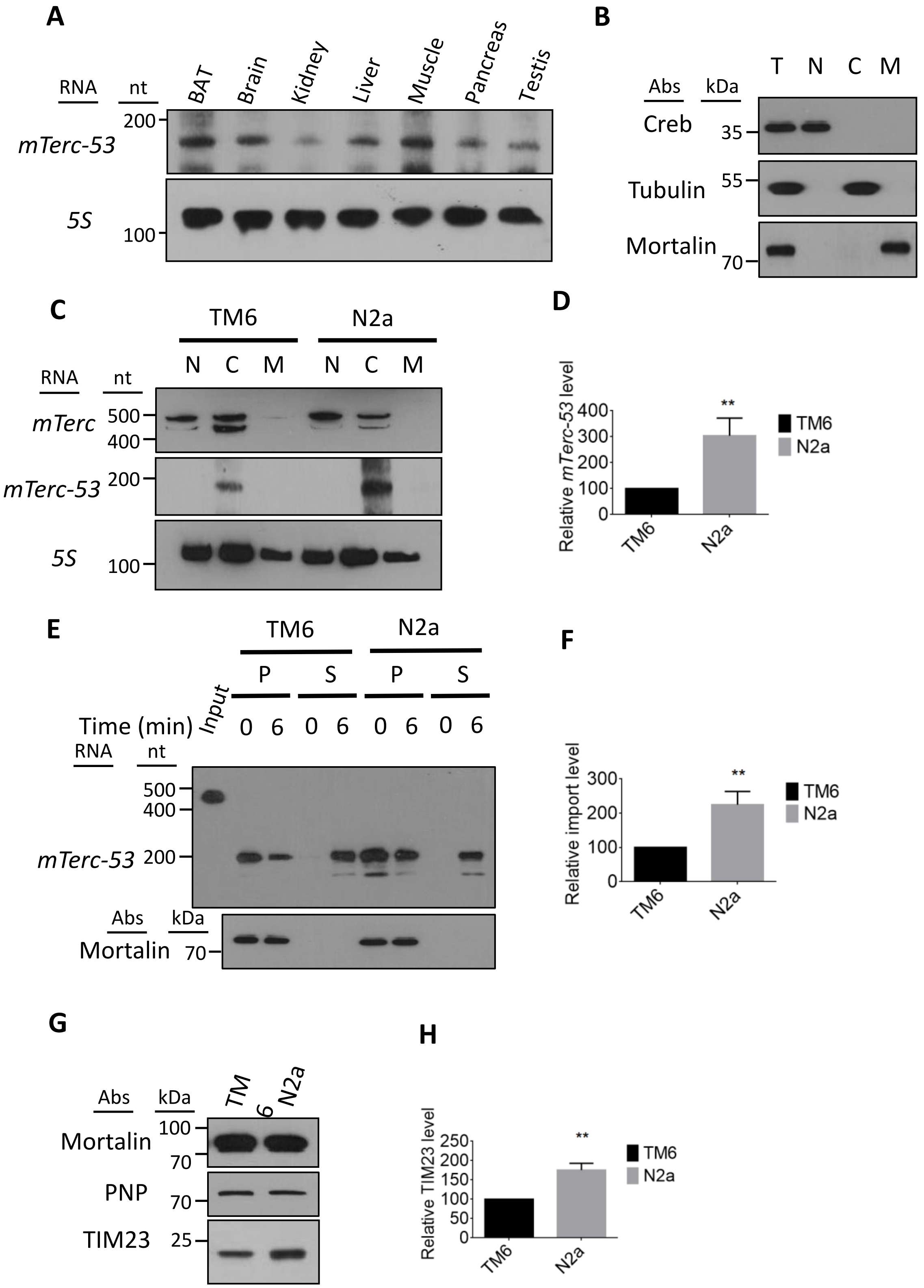
A
RNA
nt
BAT
Brain
Kidney
Liver
Muscle
Pancreas
Testis
mTerc-53
200
5S
100
B
Abs
kDa
T
N
C
M
Creb
35
Tubulin
55
Mortalin
70
C
TM6
N2a
N
C
M
N
C
M
RNA
nt
mTerc
500
400
mTerc-53
200
5S
100
D
Relative mTerc-53 level
400
300
200
100
0
**
TM6
N2a
E
TM6
N2a
P
S
P
S
Input
Time (min)
0
6
0
6
0
6
0
6
RNA
nt
500
400
mTerc-53
200
Abs
kDa
Mortalin
70
F
Relative import level
300
200
100
0
**
TM6
N2a
G
Abs
kDa
TM6
N2a
Mortalin
100
70
PNP
70
TIM23
25
H
Relative TIM23 level
250
200
150
100
50
0
**
TM6
N2a

Figure S2. *mTerc-53* levels in different mouse tissues, *mTerc-53* localization in neuronal N2a cells, in vitro import of *mTerc* into N2a mitochondria and export of *mTerc-53*, and N2a mitochondrial protein levels, related to Figure 4.

(A) Northern blots of cytosolic *mTerc-53* and *5S* rRNA in different mouse tissues. BAT (Brown Adipose Tissue). (B) Immunoblots of different cellular fractions of mouse neuronal cells N2a: total cell lysate (T), the nucleus (N), the cytosol (C) and mitochondria (M). Creb, β-tubulin, and mortalin were used as markers for the nucleus, the cytosol and mitochondria respectively. (C) Northern blots of *mTerc*, *mTerc-53* and *5S* in equal cellular volume of nuclear, cytosolic and mitochondrial fractions in mouse TM6 cells and mouse neuronal N2a cells. (D) Quantification of the relative cytosolic *mTerc-53* level in panel (C) (n = 3). (E) In vitro import of *mTerc* into TM6 or N2a mitochondria and export of *mTerc-53* from the mitochondria; P (Pellet), S (Supernatant). (F) Quantification of the relative import efficiency in panel (E) (n=3). (G) Immunoblot of TM6 and N2a mitochondria. (H) Quantification of the relative TIM23 level in panel (G) (n = 3). Statistical comparisons are performed using unpaired *t*-tests; *$P<0.05$, **$P<0.01$, ***$P<0.001$, ****$P<0.0001$. Data are presented as mean ± standard error of the mean (s.e.m.).

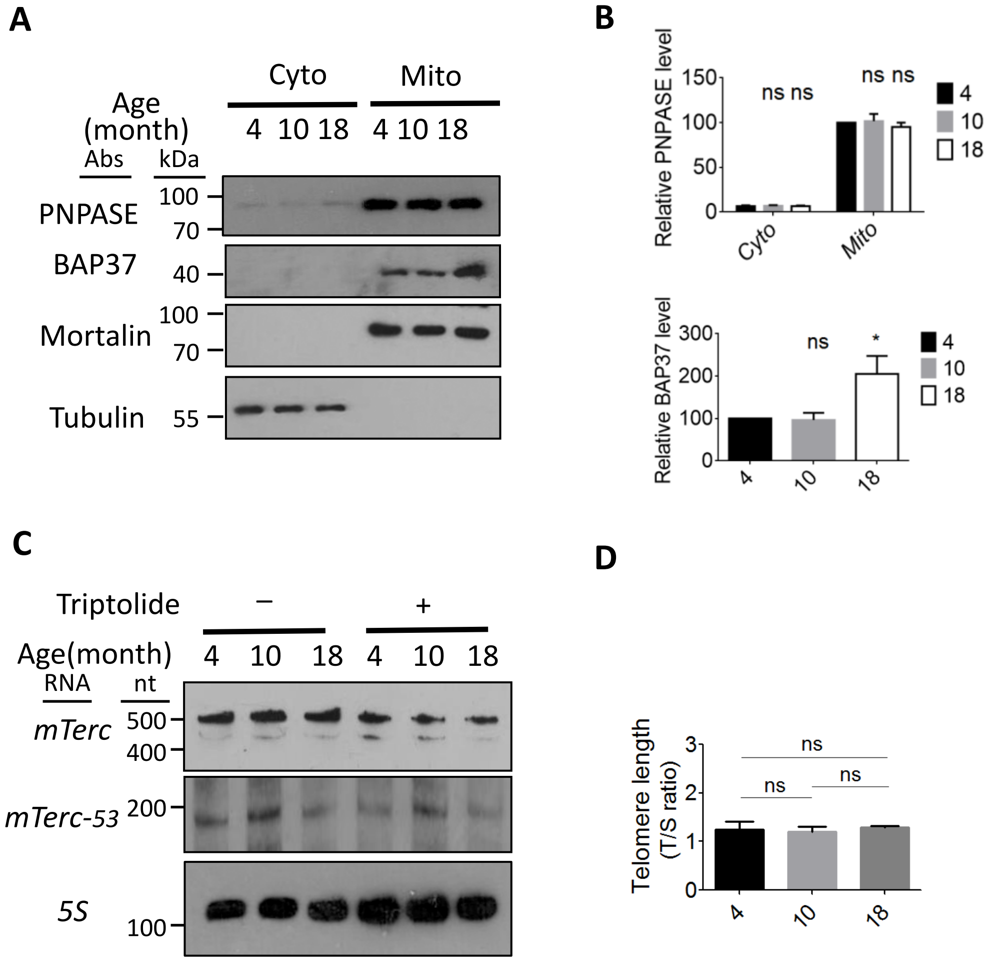

Figure S3. Protein level changes in the brains of 4 months, 10 months and 18 months old mice, and the effect of triptolide treatment on cytosolic *mTerc* and *mTerc-53* levels, related to Figure 4.

(A) Immunonlots of the cytosol and mitochondria isolated from the brains of 4 months, 10 months and 18 months old mice. (B) Quantification of the relative PNPASE levels and the mitochondrial BAP37 levels in panel (A) (n = 6). (C) Northern blots of cytosolic *mTerc*, *mTerc-53* and *5S* rRNA in the brain cells of 4 months, 10 months and 18 months old mice with or without triptolide treatment (2 μM for 3 hs). (D) Comparison of the telomere length in the brains of 4 months, 10 months and 18 months old mice. Statistical comparisons are performed using unpaired *t*-tests; **P*<0.05, ***P*<0.01, ****P*<0.001, *****P*<0.0001. Data are presented as mean ± standard error of the mean (s.e.m.).

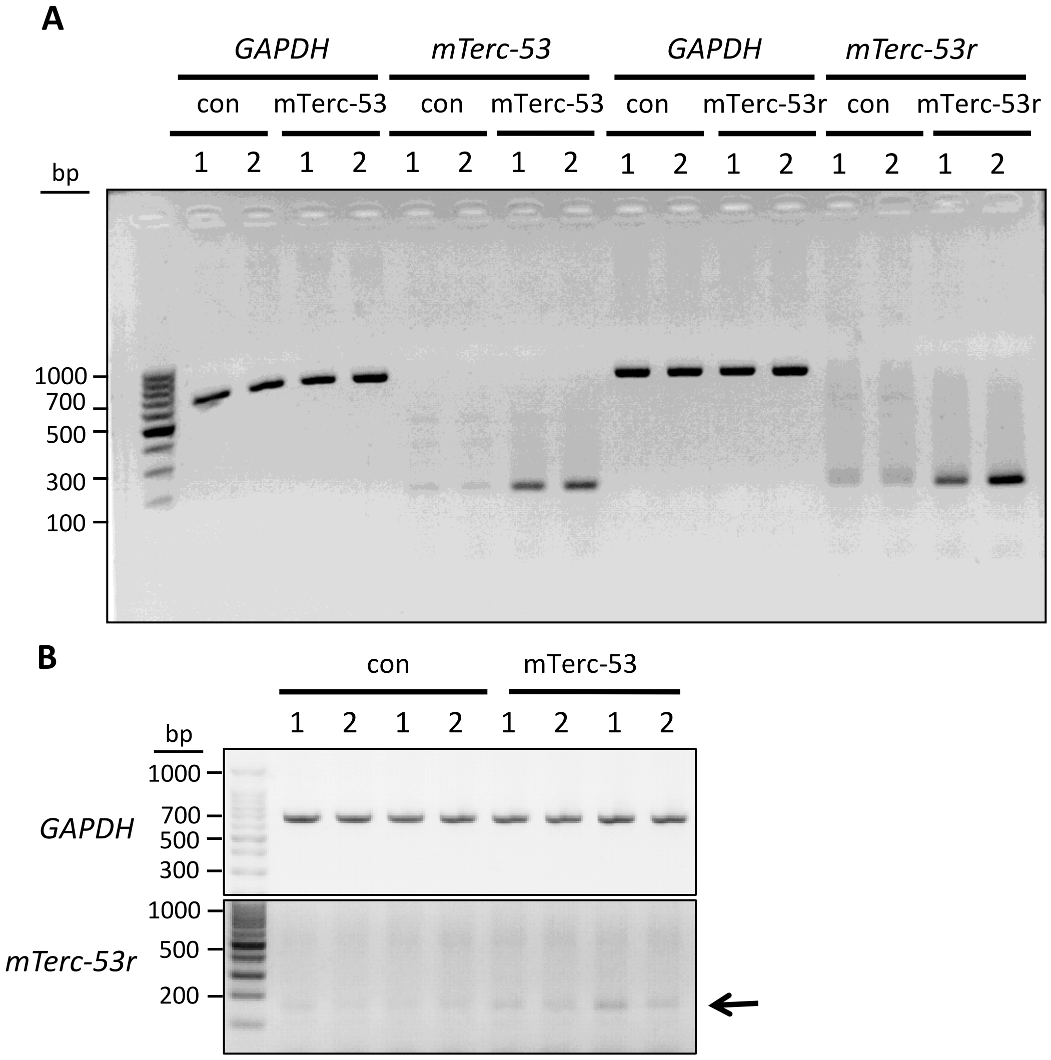

Figure S4. exogenous expression of *mTerc-53* and *mTerc-53r* in mouse hippocampi, related to Figure 4 and 5.

(A) Total RNA was isolated from *mTerc-53*, or *mTerc-53r* overexpressing mouse hippocampi or control hippocampi (con) and RT-PCR was performed with primers for *GAPDH*, *mTerc-53* or *mTerc-53r*. Two mouse hippocampi were examined (1 and 2). (B) *mTerc-53r* level was examined in mouse hippocampi with exogenous expression of *mTerc-53* or the control hippocampi.

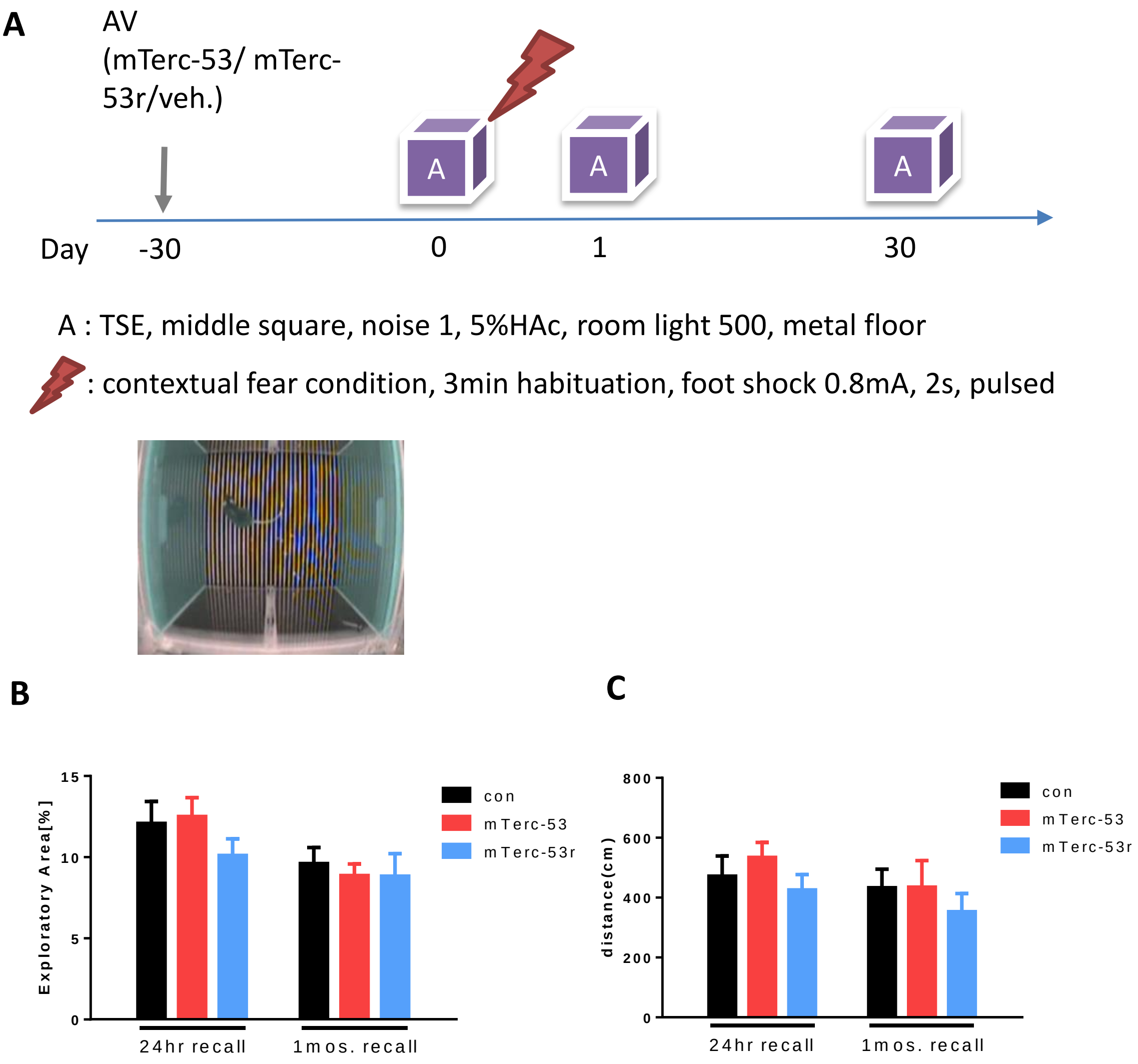

Figure S5. Contextual fear conditioning, related to Figure 4.
(A) Schematic of the contextual fear conditioning paradigm with a photo of a mouse in context A. (B) Exploratory area of control mice (n = 20) or mice overexpressing *mTerc-53* (n = 20) or *mTerc-53r* (n = 20) in the hippocampus 24 hours (24hr recall) or 1 month (1mos. recall) after training. (C) Travel distance of the mice.

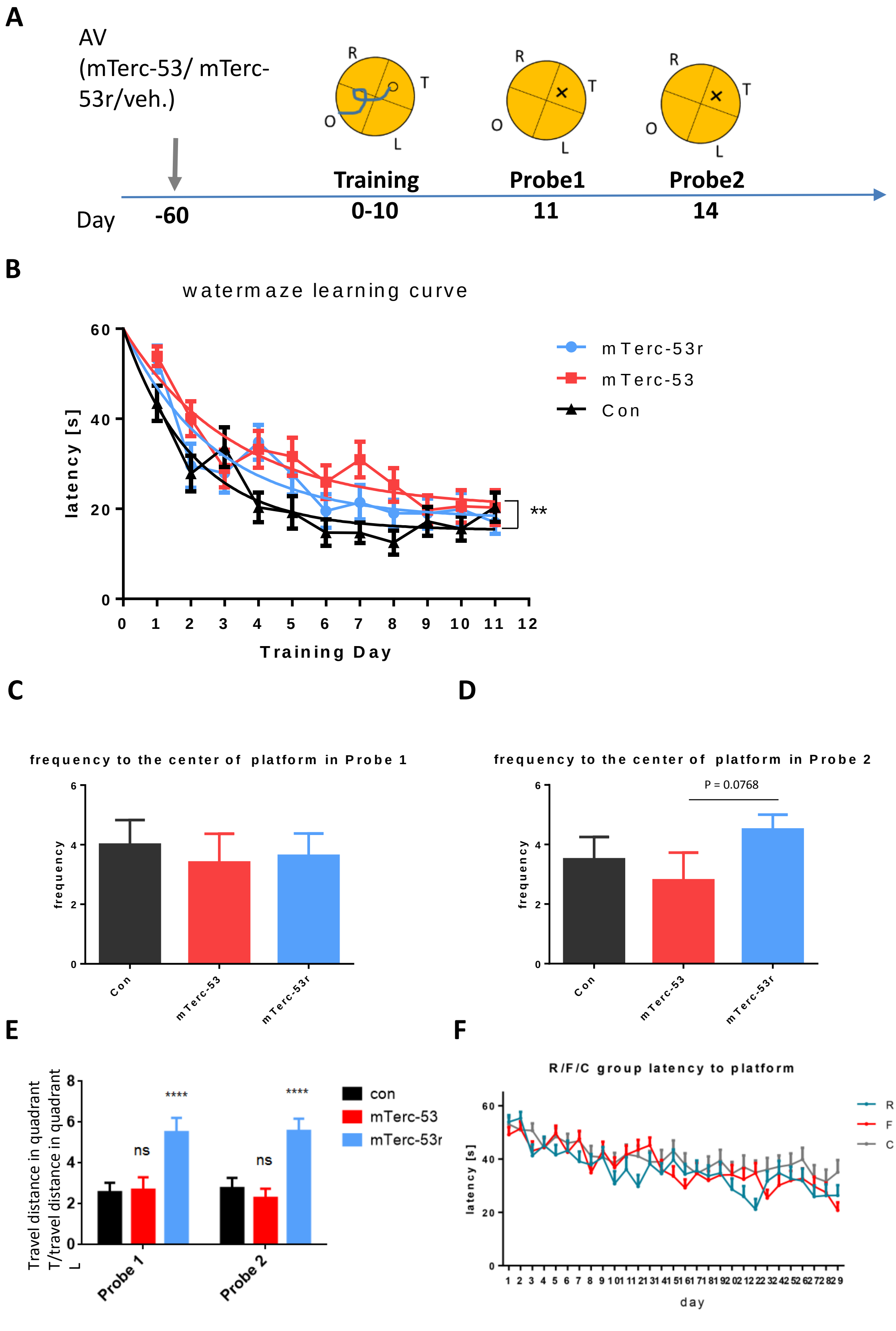
A
AV
(mTerc-53/ mTerc-53r/veh.)
R
T
O
L
Training
Probe1
Probe2
Day
-60
0-10
11
14
B
watermaze learning curve
latency [s]
Training Day
mTerc-53r
mTerc-53
Con
**
C
frequency to the center of platform in Probe 1
frequency
Con
mTerc-53
mTerc-53r
D
frequency to the center of platform in Probe 2
P = 0.0768
frequency
Con
mTerc-53
mTerc-53r
E
Travel distance in quadrant T/travel distance in quadrant L
ns
****
con
mTerc-53
mTerc-53r
Probe 1
Probe 2
F
R/F/C group latency to platform
latency [s]
day
R
F
C

Figure S6. Morris Water Maze (MWM) test, related to Figure 4.
(A) Schematic of the MWM paradigm. (B) Latency learning curve of control mice (n = 12) or mice overexpressing *mTerc-53* (n = 10) or *mTerc-53r* (n = 10) in the hippocampus to locate the hidden platform. Two-way repeated measures ANOVA was used for comparison. (C, D) Frequency of locating the hidden platform in probe 1 (C) and 2 (D). Statistical comparisons are performed using unpaired *t*-tests; (E) Ratio of travel distance in quadrant T to that in quadrant L in probe 1 and 2. (F) Latency learning curve of 18 months old control mice (C) or mice overexpressing *mTerc-53* (F) or *mTerc-53r* (R) in the hippocampus to locate the hidden platform (n = 8). Two-way repeated measures ANOVA was used for comparison.*$P<0.05$, **$P<0.01$, ***$P<0.001$, ****$P<0.0001$. Data are presented as mean ± standard error of the mean (s.e.m.).

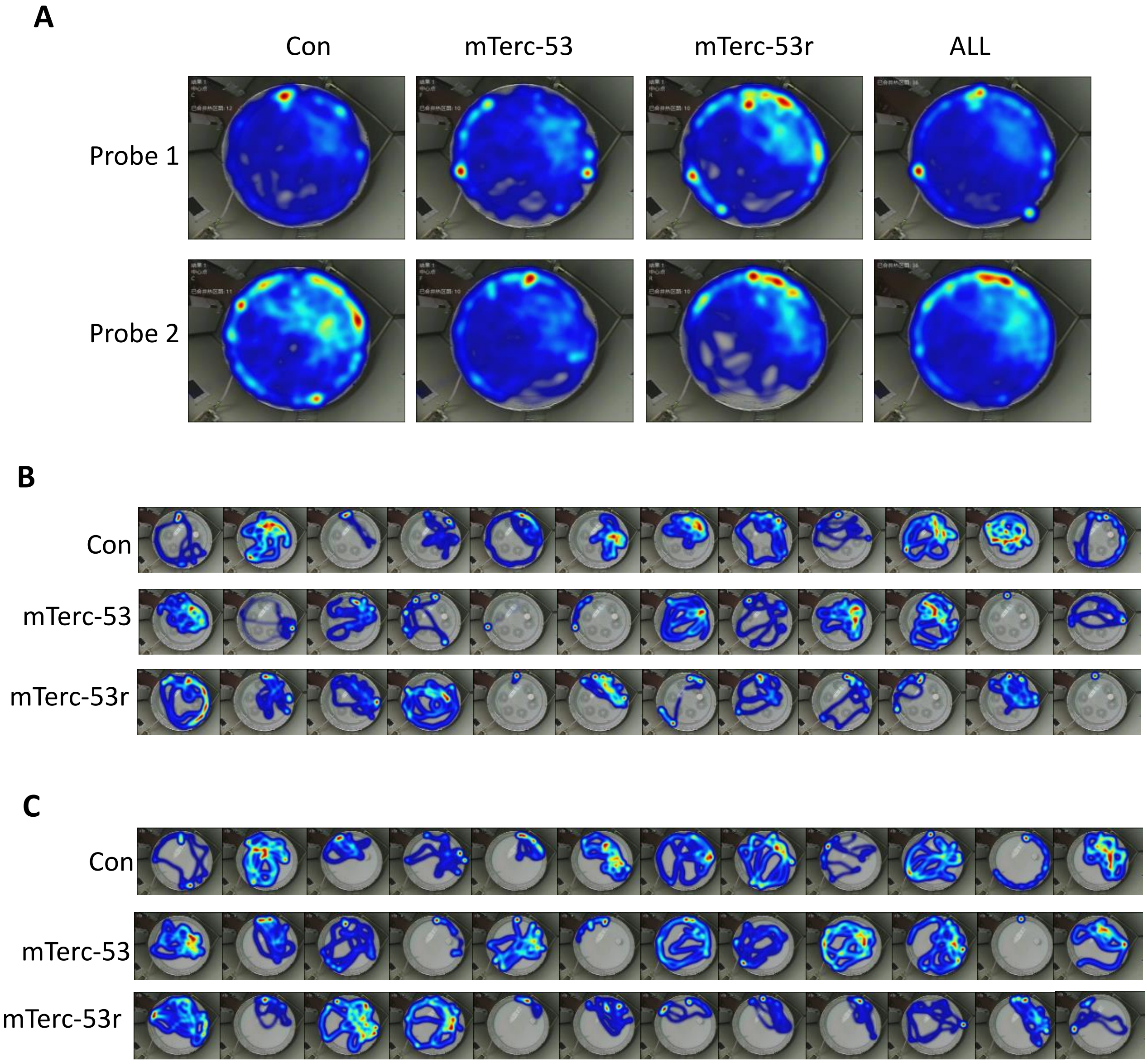

Figure S7. Morris Water Maze (MWM) test, related to Figure 4.
(A) Average traveling heat maps in three groups and in total for two probes. (B) Individual traveling heat maps during probe 1. (C) Individual traveling heat maps during probe 2.

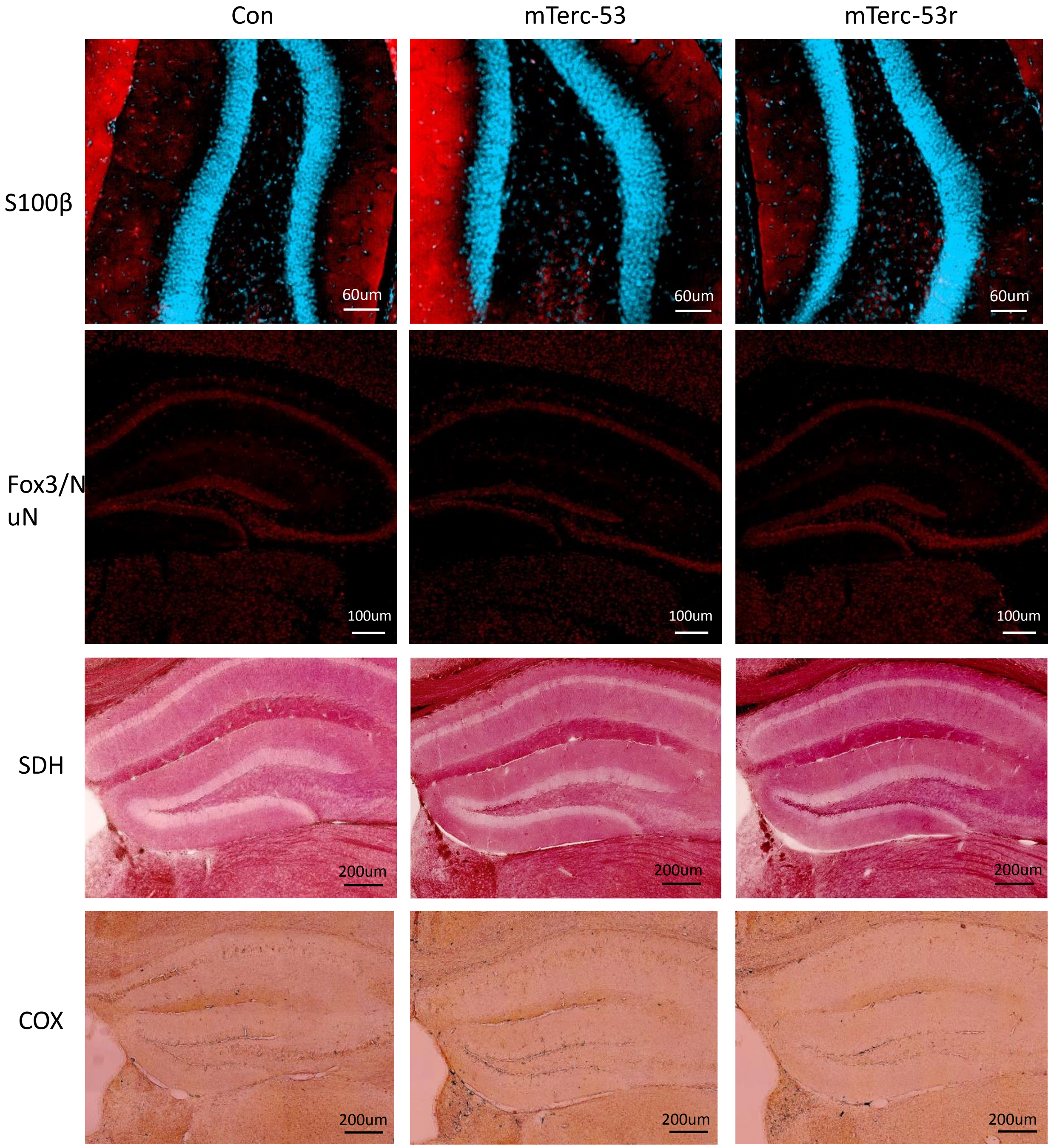

Figure S8. Histological examination of the hippocampi of control mice or mice overexpressing *mTerc-53* or *mTerc-53r,* related to Figure 5. S100β (red) and Fox3/NeuN immunostaining, and COX/SDH histochemistry of the hippocampi.

Table legends

Table S1. Differentially expressed genes between Terc-53 (53) and control cells (Con).
Table S2. Differentially expressed genes between Terc-53r (53r) and control cells (Con).
Table S3. Gene ontology of differentially expressed genes between Terc-53 (53) and control cells (Con).
Table S4. Gene ontology of differentially expressed genes between Terc-53r (53r) and control cells (Con).
